# A Mouse Model for the Metabolic Effects of the Human Fat Mass and Obesity Associated *FTO* Gene

**DOI:** 10.1371/journal.pgen.1000599

**Published:** 2009-08-14

**Authors:** Chris Church, Sheena Lee, Eleanor A. L. Bagg, James S. McTaggart, Robert Deacon, Thomas Gerken, Angela Lee, Lee Moir, Jasmin Mecinović, Mohamed M. Quwailid, Christopher J. Schofield, Frances M. Ashcroft, Roger D. Cox

**Affiliations:** 1 MRC Harwell, Metabolism and Inflammation, Harwell Science and Innovation Campus, Harwell, United Kingdom; 2 Henry Wellcome Centre for Gene Function, Department of Physiology, Anatomy, and Genetics, University of Oxford, Oxford, United Kingdom; 3 Chemistry Research Laboratory and Oxford Centre for Integrative Systems Biology, University of Oxford, Oxford, United Kingdom; 4 Department of Experimental Psychology, University of Oxford, Oxford, United Kingdom; Stanford University School of Medicine, United States of America

## Abstract

Human *FTO* gene variants are associated with body mass index and type 2 diabetes. Because the obesity-associated SNPs are intronic, it is unclear whether changes in FTO expression or splicing are the cause of obesity or if regulatory elements within intron 1 influence upstream or downstream genes. We tested the idea that FTO itself is involved in obesity. We show that a dominant point mutation in the mouse *Fto* gene results in reduced fat mass, increased energy expenditure, and unchanged physical activity. Exposure to a high-fat diet enhances lean mass and lowers fat mass relative to control mice. Biochemical studies suggest the mutation occurs in a structurally novel domain and modifies FTO function, possibly by altering its dimerisation state. Gene expression profiling revealed increased expression of some fat and carbohydrate metabolism genes and an improved inflammatory profile in white adipose tissue of mutant mice. These data provide direct functional evidence that *FTO* is a causal gene underlying obesity. Compared to the reported mouse FTO knockout, our model more accurately reflects the effect of human *FTO* variants; we observe a heterozygous as well as homozygous phenotype, a smaller difference in weight and adiposity, and our mice do not show perinatal lethality or an age-related reduction in size and length. Our model suggests that a search for human coding mutations in *FTO* may be informative and that inhibition of FTO activity is a possible target for the treatment of morbid obesity.

## Author Summary

Geneticists have identified many gene regions that cause human disease by using multiple genetic markers in large populations to find gene regions associated with disease. However, it is often not clear precisely which gene in any given region causes the disease or how the gene exerts its functional effect. For example, a gene variant in the non-coding region of FTO enhances obesity risk, but it is not clear if this is an effect of the FTO gene itself or another gene located nearby. We therefore tested whether FTO regulates body weight in the mouse. We found that a single change (mutation) in the sequence coding for the mouse FTO protein decreases the functional activity of FTO and causes reduced fat mass and body weight. Food intake and activity were normal, but the mutant mice had a higher metabolic rate. In addition, their fat mass was lower than that of normal mice when both were fed a high-fat diet. Our study provides direct evidence that FTO directly affects fat mass and thus is likely to have a role in human obesity. As reduced FTO function decreases body weight in mice, it is worth exploring if pharmaceutical agents that inhibit FTO activity might help reduce human obesity.

## Introduction

In genome-wide association studies (GWAS) for type 2 diabetes, a single nucleotide polymorphism (SNP) within intron 1 of the fat mass and obesity-associated (*FTO*) gene was found to be associated with an increased risk of obesity [1],[2]. Around 16% of the Caucasian population is homozygous for the risk allele and has an ∼1.67-fold increased risk of obesity, weighing ∼3 kg more than controls. The risk allele is not associated with fetal growth but confers an increased risk of elevated body mass index (BMI) and obesity [1] that manifests by the age of 7 and persists into adulthood. These results have largely been confirmed in other populations [2]–[7].

*FTO* is a 9-exon gene located on human chromosome 16 and mouse chromosome 8. Sequence analyses suggested that FTO has homology with the AlkB family of DNA repair enzymes. Subsequent *in vitro* biochemical studies revealed FTO to be a member of the Fe(II) and 2-oxoglutarate (2OG) dependent oxygenase superfamily [8]. In metazoans these enzymes are involved in diverse processes including oxygen sensing, DNA repair, fatty acid metabolism and post-translational modifications [9]. Recombinant FTO catalyses oxidative demethylation of 3-methylthymine and 3-methyluracil in single-stranded DNA and RNA [8],[10], suggesting its physiological role involves nucleic acid modification. *In vitro* expression of murine FTO results in localisation of the recombinant protein to the nucleus, consistent with a role in nucleic acid modification [8].

FTO is ubiquitously expressed. In the brain, mRNA levels are particularly high within the hippocampus, cerebellum and hypothalamus [8],[11]. In the hypothalamus, strong expression is seen in the arcuate, paraventricular, dorsomedial and ventromedial nuclei - sites critical for regulating energy balance [12].

The hypothalamic expression of *FTO* suggests a potential role in the control of food intake and whole body metabolism. Consistent with this idea, several studies have reported higher levels of energy intake in individuals with the at-risk *FTO* allele [13]–[19]. Although other studies have found no association of variants with energy expenditure measured by calorimetry in adults [16],[20] or food intake [21], the balance of evidence from population-based studies suggests that *FTO* intron 1 SNPs are associated with increased energy intake.

GWAS are a powerful way of identifying genes involved in common disease but it is often difficult to translate their findings into an understanding of how the gene products act at the cellular and whole animal levels. This is a particular problem when the disease-associated SNP lies within an intron, as is the case for *FTO*. One approach to identifying the function of a gene is to mutate it in a model organism. A mouse possessing a 1.6 Mbp deletion in mouse chromosome 8 that includes *Fto* as well as *Ftm*, *Ft1*, *Irx3*, *Irx5* and *Irx6* has been characterised (*fused toes* or *Ft* mice) [22]. Homozygous *Ft* mice are embryonic lethal and display neural tube defects, left-right asymmetry and polydactyly. Heterozygous mice have fusion of the forelimb digits and thymic hyperplasia [23]. Recently, a mouse FTO knockout has been described in which exons 2 and 3 of *Fto* are replaced by a neomycin STOP cassette [24]. This cassette also deletes part of intron 1 although not the position equivalent to the BMI-associated SNPs in human *FTO*. Homozygous FTO knockout mice were viable but showed significant postnatal death before 4 weeks of age. They also exhibited postnatal growth retardation, a reduction in adipose tissue, a reduction in lean mass, increased energy expenditure, increased sympathetic nervous system activity, relative hyperphagia and reduced spontaneous locomotor activity. Thus this study suggested that *Fto* is directly involved in energy metabolism and body weight regulation.

However, many human SNPs are likely to result in impaired function rather than total loss of function, and mouse knockouts are not always the most informative because they can result in a non-viable or severely compromised organism. Indeed, knockout of the mouse *Fto* gene is associated with increased postnatal death and growth retardation [24], which is not observed for human *FTO* variants. We therefore searched the Harwell mouse *N-*ethyl-*N*-nitrosourea (ENU) archive for an *Fto* mutation that produces a partial loss of function.

We describe here a dominant missense mutation (I367F) in the mouse *Fto* gene that results in a lean phenotype of reduced body weight and fat mass. Physical activity and food intake is unchanged but metabolic rate is increased. We show by gene expression analysis that fat and carbohydrate metabolism are increased in mutant mice and they possess an improved inflammatory profile. On a high-fat diet mutant mice show a lower fat mass than wild-type mice. These results indicate that our mice provide an improved model for the human phenotype and provide functional evidence that the *FTO* gene is a causal gene underlying obesity.

## Results

### A Missense Mutation within the *C*-terminal Domain of FTO

**Figure 1:**
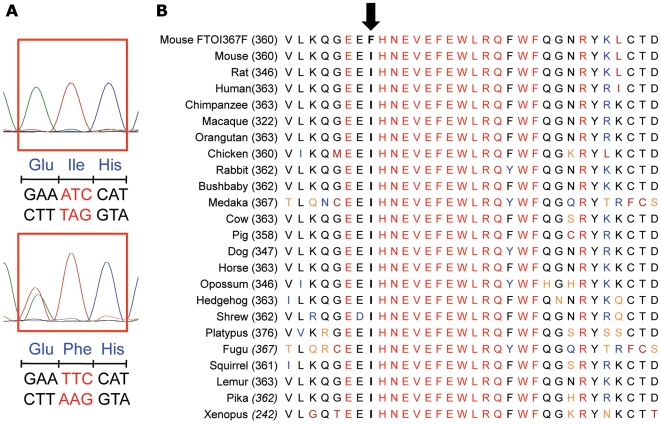
An ENU-induced missense mutation I367F in exon 6 of FTO. (A) Sequencing reveals an A to T mutation in exon 6 of FTO that leads to conversion of the isoleucine at residue 367 to phenylalanine. (B) Sequence line-up showing isoleucine 367 (arrow) and surrounding residues are highly conserved in vertebrates. Colour code for amino acids indicates a consensus residue derived from a block of similar residues (black), a completely conserved residue (red), a residue weakly similar to a consensus residue (orange), a consensus residue derived from the occurrence of greater than 50% of a single residue (blue) and non-similar residues (dark-red).

In a screen of DNA from 6,624 mice from the Harwell ENU-induced mutagenesis archive [25] we identified an adenosine-to-thymidine mutation in exon 6 of *Fto* leading to substitution of a phenylalanine for isoleucine-367 (*Fto^I367F^*) in the *C*-terminal region of murine FTO (mFTO) (Figure 1A). Although this substitution lies outside the predicted double-stranded β-helix (DSBH) and associated elements that form the conserved “catalytic core” of 2OG oxygenases [9], the block of ∼20 amino acids surrounding I367 is conserved in FTO throughout vertebrate evolution (Figure 1B), suggesting this region has a physiological role.

The *C*-terminal regions of some 2OG oxygenases are known to facilitate oligomerisation [9],[26]. We therefore compared expression of full-length and *C*-terminal truncated (residues 1–408, 1–387 and 1–329 inclusive) mFTO in *Escherichia coli*. Although wild-type mFTO was efficiently produced in a soluble form, *C*-terminally truncated forms of wild-type mFTO and full-length mFTO^I367F^ gave only low amounts of largely insoluble protein (data not shown). By contrast, a His-tagged version of the *C-*terminal domain alone (residues D329-end) of wild-type mFTO (CmFTO) was efficiently produced in soluble form, CmFTO^I367F^ was largely insoluble. Mutation of residue I367 to alanine (I367A), however, yielded soluble mFTO^I367A^ and CmFTO^I367A^ proteins.

**Figure 2:**
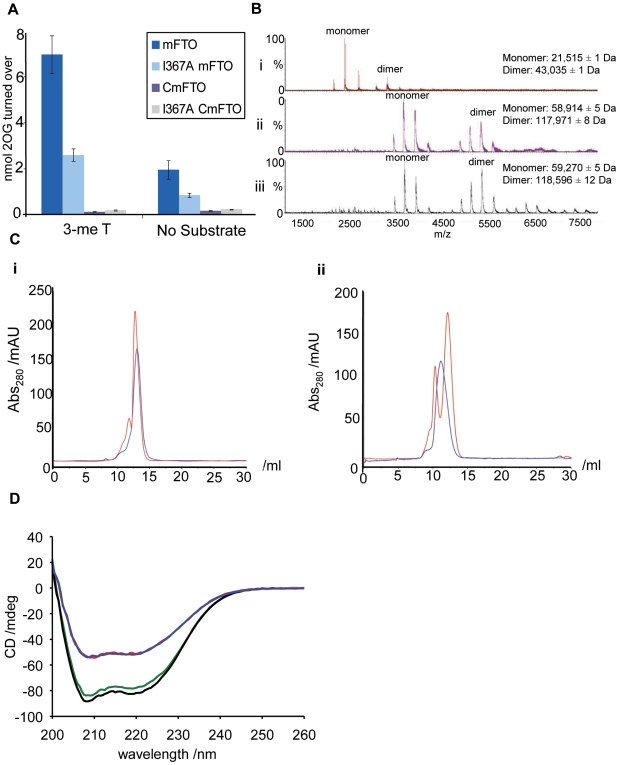
The FTO^I367F^ mutation is within a *C-*terminal domain. (A) mFTO^I367A^ (light blue) shows reduced catalytic activity in a 2OG turnover assay with a 3-meT oligonucleotide substrate, compared to mFTO (dark blue). Neither CmFTO (purple) nor CmFTO^I367A^ (grey) show significant 2OG turnover. Data are expressed as mean±SEM. (B) Non-denaturing electrospray ionisation mass spectrometry analyses showing the dimeric nature of (i) The *C-*terminal domain of mFTO (CmFTO), (ii) Full-length mFTO and (iii) Full-length human FTO. The twin peaks present in the CmFTO sample likely result from α-N-gluconylation of the protein resulting in partial formation of a+178Da adduct (see Figure S1 and Figure S2). (C) Analytical gel filtration chromatogram showing the presence of two FTO species in solution. (i) mFTO (red) and mFTO^I367A^ (blue), (ii) CmFTO (red) and CmFTO^I367A^ (blue). (D) Circular dichroism spectra showing the secondary structures of mFTO (red), mFTO^I367A^ (blue), CmFTO (green) and CmFTO^I367A^ (black) at 0.25 mg/ml, 4°C.

The catalytic activity of mFTO^I367A^ in the presence of a 3-methylthymine containing oligonucleotide substrate, as measured by 2OG turnover, was only ∼40% of wild-type mFTO (Figure 2A). As anticipated, because they lack the DSBH core, neither CmFTO nor CmFTO^I367A^ were catalytically active (Figure 2A).

We analysed the oligomerisation states of mFTO and human FTO (hFTO) by non-denaturing mass spectrometry (MS, Figure 2B) and found that mFTO, hFTO and CmFTO exist as a mixture of monomeric and dimeric forms (Figure S1 and Figure S2). However, the proportion of dimeric mFTO^I367A^ is significantly reduced relative to that in the wild-type mFTO (Figure S3). We also investigated the oligomerisation state of the proteins in solution using gel filtration chromatography (Figure 2C). This revealed that whereas wild-type mFTO and CmFTO exist in both monomeric and dimeric forms, both I367A variants exist, at least predominantly, as monomers. These results suggest that I367 is involved in dimerisation of FTO (Figure 2C). It was not possible to analyse mFTO^I367F^ or CmFTO^I367F^ because of their insoluble nature.

The secondary structure of the *C*-terminal domain was then investigated using circular dichroism spectroscopy (Figure 2D). In contrast to the full-length proteins, which contain the DSBH “core” domain of the 2OG oxygenases (comprising 8 β-strands with surrounding loops and α-helices), the *C*-terminal domain proteins are predominantly α-helical (Figure S4 and Figure S5). A similar structural difference was observed for hFTO and its *C*-terminal domain (residues 332-end; ChFTO, Figure S6). The I367A mutation does not appear to alter the overall secondary structure fold of mFTO proteins (Figure 2D), despite preventing dimerisation. This suggests that the I367A proteins are correctly folded, but that isoleucine 367 is involved in dimer formation. Factor Inhibiting Hypoxia Inducible Factor (FIH), a human 2OG-oxygenase involved in the hypoxic response, also exists as a dimer [26]. For FIH, dimerisation is enabled by a *C*-terminal region. However, sequence comparisons, coupled with structural predictions, suggest that the *C*-terminal region of FTO and FIH will be substantially different, with that of FTO having a more α-helical character.

Overall, these results reveal that FTO can exist in both monomeric and dimeric forms and that the *C*-terminal domain is involved in dimerisation. The evidence suggests that the I367A (and presumably I367F) mutation disrupts dimerisation and results in a reduction of catalytic activity. In addition to the presumed lower activity of mFTO^I367F^, we cannot exclude the possibility that a reduced stability of mFTO^I367F^, leading to aggregation and/or proteolytic degradation, contributes to the phenotype.

### The Mutant FTO Protein Is Localised to the Nucleus and Expressed at Lower Levels in Mammalian Cells

**Figure 3:**
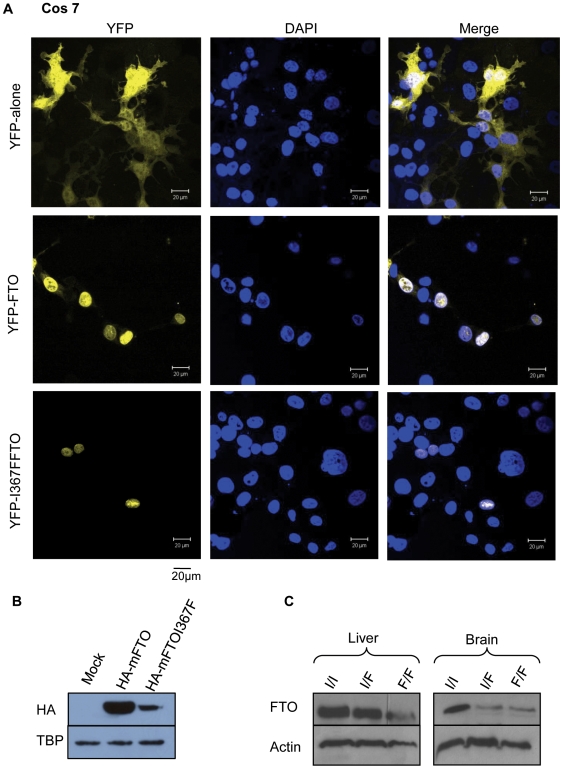
The FTO^I367F^ protein is expressed at lower levels and localised in the nucleus. (A) Confocal fluorescence images of COS-7 cells expressing YFP, YFP-FTO and YFP-FTO^I367F^, as indicated. First column: YFP fluorescence. Second column: fluorescence from 4′,6-diamidino-2-phenylindole (DAPI), which stains nuclei. Third column: merged channels. (B) Western blot of protein from Cos cells expressing HA-FTO or HA-FTOI367F, as indicated, probed with an anti-HA antibody. Below, loading control of the same gel probed with an anti-TATA-binding protein (TBP) antibody. (C) Western blot of protein from liver and brain of wild-type (I/I), heterozygous (I/F) and homozygous (F/F) mice probed with a polyclonal rabbit anti-FTO antibody. Below, loading control of the same gel probed with an anti-actin antibody.

To establish if mFTO^I367F^ is stable in mammalian cells we transfected Cos-7 and PC12 cell lines with a plasmid encoding *Fto* or *Fto^I367F^ N*-terminally tagged with yellow florescent protein (YFP), or with YFP alone. Imaging revealed that like mFTO [8], mFTO^I367F^ localised to the nucleus (Figure 3A and data not shown). However, fewer YFP-positive cells were observed than when mFTO was transfected, suggesting mFTO^I367F^ is expressed at lower levels. This was confirmed by immunoblotting of lysates from cells expressing HA-tagged wild-type or mutant FTO (Figure 3B). Similarly, immunoblotting of heterozygous and homozygous *Fto^I367F^* mouse brain and liver with an FTO antibody confirmed that the mutant protein is expressed at lower levels than wild-type (Figure 3C). These data therefore suggest that the I367F mutation results in reduced FTO protein levels and thus is likely to impair FTO function. However, it does not produce a total knockout as some mFTO^I367F^ protein appears to be expressed and correctly targeted to the nucleus.

### *Fto^I367F^* Mice Are Lean

**Figure 4:**
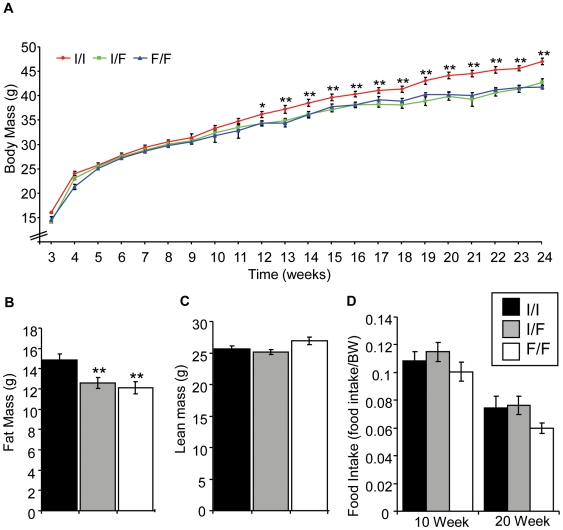
Male *Fto^I367F^* mice exhibit reduced body and fat mass. (A) Body weights of male heterozygous (I/F; n = 21) and homozygous (F/F; n = 28) *Fto^I367F^* mice and of wild-type littermates (I/I; n = 15). (B,C) Fat mass (B) and lean body mass (C) of 24-week old male heterozygous (n = 26), homozygous (n = 15) and wild-type (n = 12) mice. (D) Food intake over 24 hours normalised to body weight (BW) measured at 10 weeks (I/I, n = 20; I/F, n = 30; F/F, n = 15) and 20 weeks (I/I, n = 12; I/F, n = 20; F/F, n = 15). Data are expressed as mean±SEM, Statistical analysis was performed using Student's t-test. *, P<0.05; **, P<0.01 for differences between *Fto^I367F^* heterozygous or homozygous mice and wild-type littermates.

In order to determine if the mutation affects body mass, mice were weighed and measured weekly from 3 to 24 weeks. Both homozygous and heterozygous male mice carrying the dominant *Fto^I367F^* mutation exhibited a maturity-onset reduction in body weight (Figure 4A) diverging from wild-type at 12 weeks of age (P = 0.01) and becoming ∼10% less than wild-type by 24 weeks. There was no significant difference in body weight between heterozygous and homozygous male mice. No difference was observed between wild-type and mutant female mice (Table S1), and all further experiments described were therefore confined to male mice.

Dual Energy X-ray absorptiometry (DEXA) analysis revealed a 16–18% reduction in total fat mass between 24-week old *Fto^I367F^* and wild-type mice (Figure 4B). The proportion of body mass in fat was reduced by ∼4–7% from 36.4% fat in wild-type to 30.8–32.9% fat in mutant mice. No significant difference in lean body mass was observed (Figure 4C). This suggests that the lower weight of the mutant mice is largely attributable to a decrease in fat mass. Measurement of 24-hour cumulative food intake revealed no statistically significant difference between control and *Fto^I367F^* mice on a normal chow diet at either 10 or 20 weeks, even after normalisation for body weight (Figure 4D).

### Mice Carrying the *Fto^I367F^* Mutation Have a Higher Metabolic Rate

**Figure 5:**
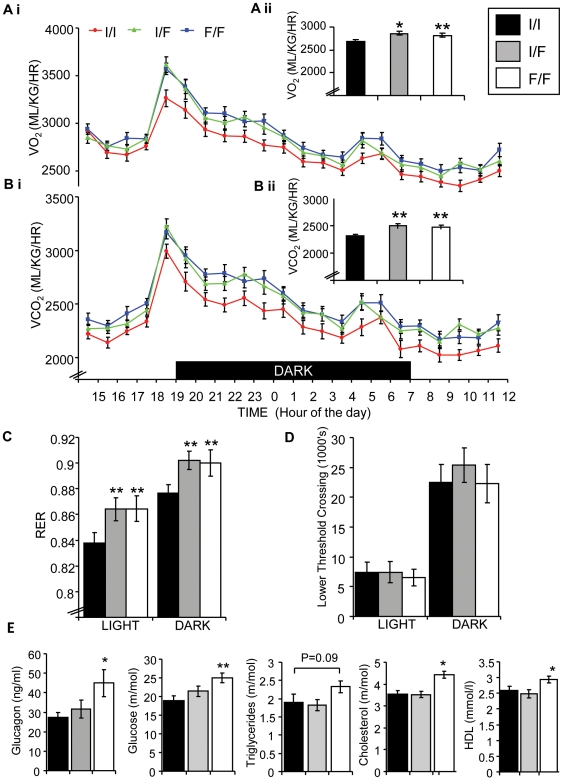
*Fto* ^I367F^ mice exhibit enhanced metabolism. (A,B) Oxygen Consumption (A i) and carbon dioxide production (B i) over a 22-hr period for 18-week old male heterozygous (I/F; n = 30) and homozygous (F/F; n = 15) *Fto^I367F^* mice and wild-type littermates (I/I; n = 26). Inset, total oxygen consumption (A ii), and carbon dioxide production (B ii) over 22-hr. (C) Respiratory exchange ratio (RER) calculated from data given in A, B during the light and dark period. (D) Physical activity over 23-hr for 18-week old male heterozygous (n = 10), homozygous (n = 11) and wild-type (n = 7) littermates. (E) Glucagon, glucose and lipid serum levels (as indicated) measured after overnight fasting at 24-weeks of age. WT (I/I; n = 12), heterozygous (I/F; n = 17), and homozygous (F/F; n = 15) littermates. Data are expressed as mean±SEM. *, P<0.05; **, P<0.01; ***, P<0.001 (Student's t-test) for differences between *Fto^I367F^* and wild-type mice.

To investigate the mechanism underlying the reduced body weight, we measured O_2_ consumption, CO_2_ production and respiratory exchange ratio (RER) at 18 weeks of age. Heterozygous and homozygous *Fto^I367F^* mice showed an increase in O_2_ consumption (Figure 5A) and CO_2_ production (Figure 5B) during both the light and dark phase of activity. As a consequence, RER was increased (Figure 5C). These data indicate the *Fto^I367F^* mutation causes an increase in whole body metabolism and a switch to relatively more carbohydrate metabolism. There was no difference in physical activity between wild-type and *Fto^I367F^* mice, as assessed by threshold crossing (Figure 5D), during either the light and dark periods of a 22-hour measurement period.

### *Fto^I367F^* Mice Show an Altered Leptin-Adipose Mass Set-Point and Increased Fasted Glucagon at 24 Weeks

An intraperitoneal glucose tolerance test (IPGTT) at 12 and 16 weeks of age did not reveal a significant difference between *Fto^I367F^* and wild-type mice (Text S1 and Figure S7A, S7B). Fasted serum leptin levels were also not significantly different at 24 weeks (Figure S7C). When circulating leptin levels were plotted against the percentage of body fat we found (as expected) a positive correlation for both heterozygous and homozygous *Fto^I367F^* mice (r 0.56, r^2^ 0.31, P = 0.0295 and r 0.54, r^2^ 0.30, P = 0.0238, respectively). However, circulating leptin as a function of percentage body fat in homozygotes was shifted to the left in comparison to controls suggesting higher leptin secretion per unit of body fat (Figure S7D). Levels of fasting serum glucagon, triglycerides, cholesterol and HDL were increased in 24-week old *Fto^I367F^* homozygous mice (Figure 5E). Fasting serum glucose was also increased at 24 weeks (Figure 5E) but not at 12 or 16 weeks of age (Figure S7A, S7B). No significant difference was observed in serum insulin or adiponectin (Figure S7E, S7F). A 6-hour cold challenge at 22 weeks did not reveal a significant difference between genotypes (data not shown) suggesting that thermoregulation by brown adipose tissue (BAT) is unaltered.

### *Fto^I367F^* Mice Have Altered Catecholamines and β-3 Adrenoreceptor Expression

**Figure 6:**
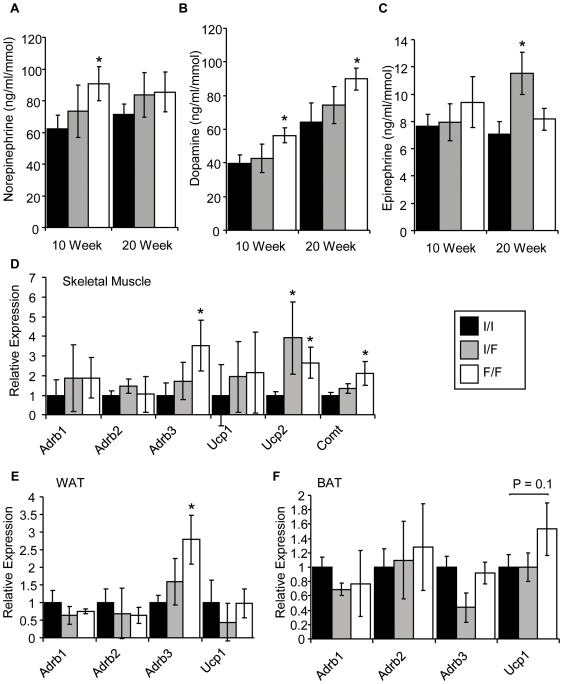
*Fto^I367F^* increases sympathetic activity. (A–C) Urinary catecholamines norepinephrine (A), dopamine (B) and epinephrine (C) at 10 and 20 weeks (10W; 20W) in heterozygous (I/F; grey bars) and homozgygous (F/F; white bars) *Fto^I367F^* mice compared to wild-type controls (I/I; black bars). 10 weeks: I/I, n = 13; I/F, n = 10; F/F = 10. 20 weeks: I/I, n = 14; I/F, n = 14; F/F, n = 15. (D–F) Gene expression in skeletal muscle (D), WAT (E) and BAT (F) expressed relative to GAPDH. Abbreviations not used previously: adrenergic beta-1 receptor (Adrb1), adrenergic beta-2 receptor (Adrb2), uncoupling protein 1 (Ucp1). Data are expressed as mean±SEM. *, P<0.05 (Student's t-test) for differences between *Fto^I367F^* and wild-type mice.

Urinary catecholamines were analysed at 10 and 20 weeks of age and normalised to urinary creatinine to control for volume. Ten-week old homozygous *Fto^I367F^* mice showed significantly higher norepinephrine (Figure 6A) and dopamine (Figure 6B), but no difference in epinephrine (Figure 6C), compared to wild-type mice. However, only dopamine was elevated in 20-week old homozygous *Fto^I367F^* mice (P<0.05).

Sympathetic stimulation of white adipose tissue (WAT), brown adipose tissue (BAT) and muscle increases metabolism by stimulating lipolysis and thermogenesis. Expression of β-3 adrenoreceptor mRNA was increased in muscle and WAT, but not BAT, of 16-week old homozygous *Fto^I367F^* mice as measured by quantitative reverse transcriptase-polymerase chain reaction (qRT-PCR) (Figure 6D–6F). Expression of uncoupling protein 2 (*Ucp2*), and the catecholamine degradation enzyme catechol-O-methyl transferase (*Comt*) were also upregulated in muscle of homozygous *Fto^I367F^* (Figure 6D).

### *Fto^I367F^* Mice Have Reduced Fat Mass and Increased Lean Mass on a High-Fat Diet

Analysis of 24-week-old mice given a high-fat diet from weaning revealed a significant reduction in fat mass and increased lean tissue mass in *Fto^I367F^* mice compared with wild-type mice (Figure S8A, S8B, S8C). There was no difference in food intake between genotypes at 10 and 20 weeks (Figure S8D). Fasting serum glucagon, glucose, triglycerides, and HDL-C levels were all increased in *Fto^I367F^* homozygous mice whereas insulin levels were significantly lower (Figure S8E, S8F, S8G, S8H). Fasted serum leptin levels were significantly increased at 8 weeks in *Fto^I367F^* mice but not at 24 weeks.

A high-fat diet significantly increased oxygen consumption in both wild-type and mutant mice (Figure S8I, S8J, S8K, S8L, S8M), whereas carbon dioxide production increased in wild-type but not *Fto^I367F^* mice (Figure S8J, S8M). Interestingly, we did not observe significant differences in either oxygen consumption or carbon dioxide output between mutant and wild-type littermates on a high-fat diet (in contrast to a normal diet). The RER of all mice was significantly reduced on a high-fat diet, reflecting an increase in relative fat metabolism. In the dark phase (when mice are active) *Fto^I367F^* mice maintained a significantly higher RER than wild-type mice (Figure S8K).

### Gene Expression Analysis in *Fto^I367F^* Mutants

**Figure 7:**
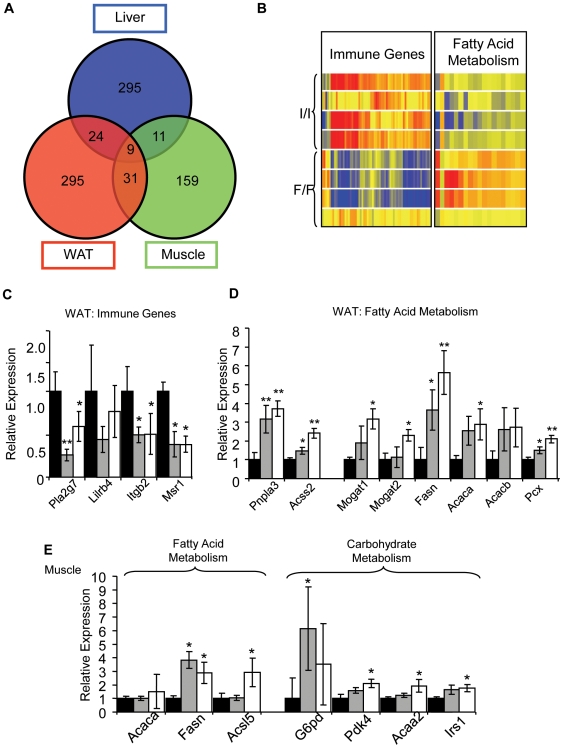
*Fto^I367F^* gene expression analyses. (A) Overlap between statistically changing genes with≥1.5 fold change in WAT, liver and skeletal muscle of homozygous *Fto^I367F^* mice. (B) Hierarchical clustering of 121 differentially expressed immune genes and 22 fatty acid metabolism genes. Each row represents an individual animal and each column a single gene. Red-blue color code represents the relative intensity of the expression signal, with red indicating high expression and blue low expression. (C–E) Gene expression levels, relative to GAPDH, measured by q-RT-PCR for immune genes in WAT (C), and genes involved in fatty acid metabolism (D), or metabolism (E) in skeletal muscle. Black bars: wild-type mice (I/I; n = 10). Grey bars: heterozygous mice (I/F; n = 9). White bars: homozygous (F/F; n = 11). Abbreviations not used previously: *Acsl5*, acyl-CoA synthetase long-chain family member 5; *Acaa2*, acetyl-Coenzyme A acyltransferase 2. Data are expressed as mean±SEM. *, P<0.05. **, P<0.01. ***, P<0.001 (Student's t-test) for differences between *Fto^I367F^* and wild-type mice.

To determine if gene expression was altered in *Fto^I367F^* mice we carried out microarray analysis of WAT, liver and skeletal muscle from 16-week old wild-type, heterozygous and homozygous *Fto^I367F^* mice. The genes that changed in heterozygous mice were largely a subpopulation of those seen to change in homozygous mice: thus we focus here on differences between homozygous and wild-type animals. Significant gene expression changes were found in all tissues, but very few were present in more than one tissue. Only 9 genes were found to change in all three tissues, 11 were common to muscle and liver, 24 to liver and WAT, and 31 to muscle and WAT (Figure 7A). Few of these changes were obviously related to the lean phenotype.

Multiple genes involved in inflammation were markedly down-regulated in abdominal WAT (Figure 7B and Table S2). These include the phospholipase A2 *Pla2g7* (6.2-fold), the leukocyte immunoglobulin-like receptor *Lilrb4* (4.7-fold), the Integrin beta 2 *Itgb2* (4.3fold), the macrophage scavenger receptor 1 *Msr1* (3.6 fold), and very many more. A reduction in expression of genes involved in the inflammatory response is consistent with the lean phenotype of the *Fto^I367F^* mice as adipose tissue normally contains resident macrophages [27],[28],[29]. The array results were in good agreement with those obtained by qRT-PCR analysis (Figure 7C).

Significantly, genes involved in the regulation of fat metabolism were also altered. Some genes involved in fatty acid catabolism were upregulated in WAT, as assessed by both microarray and qRT-PCR (Figure 7B and 7D and Table S3). These include patatin-like phospholipase domain containing 3 (*Pnpla3*), a triacylglycerol lipase, and acyl CoA synthetase (*Acss2*). Such changes may help explain the lower fat mass and higher RER of *Fto^I367F^* mice. Interestingly, *Pnpla3* has been associated with fatty liver disease in humans [30]: however the liver triglyceride concentration was unchanged in *Fto^I367F^* mice (Figure S7G). Genes involved in fatty acid synthesis were also upregulated, including fatty acid synthase (*Fasn*), pyruvate carboxylase (*Pcx*), monoacylglycerol O-acyltransferase (*Mogat1*, *Mogat2*), and acetyl-Coenzyme A carboxylase (*Acaca*, *Acacb*). Given the reduced amount of WAT in *Fto^I367F^* mice, upregulation of genes involved in fat synthesis may be a secondary adaptation that attempts to compensate for the lower fat levels.

In muscle, fatty acid synthase (*Fasn*) was strongly upregulated in 16- week old homozygous *Fto^I367F^* mice, being 10-fold higher by microarray and 3.5-fold higher by qRT-PCR (Figure 7E). Some genes involved in carbohydrate catabolism were also slightly upregulated, including glucose-6-phosphate dehydrogenase (*G6pd*), pyruvate dehydrogenase kinase (*Pdk4*) and insulin receptor substrate 1 (*Irs-1*; Figure 7E, Table S4). These changes are consistent with the idea that carbohydrate metabolism may be increased in muscle of *Fto^I367F^* mice.

Genes associated with endoplasmic reticulum (ER) stress and the unfolded protein response (UPR) also showed a small increase (≥1.3-fold) in expression in the liver of 16-week old homozygous *Fto^I367F^* mice, as assessed by microarray studies (Figure S9). Such changes are too small to be measured by qRT-PCR. There were no major changes in metabolic genes, consistent with the lack of a change in plasma glucose concentrations at this time point.

qRT-PCR analysis of the key hypothalamic neuropeptides *Agrp*, *Npy* and *Pomc* revealed no significant changes in relative mRNA expression between *Fto^I367F^* and wild-type mice in the fasted state (Figure S10). However, a significant reduction in *Npy* expression was observed in *Fto^I367F^* mice in the free-fed state (Figure S10).

## Discussion

We have identified a point mutation in the mouse *Fto* gene at a highly conserved residue within a novel *C-*terminal domain that is largely composed of α-helices and is involved in FTO dimerisation. Like the wild-type protein, the FTO^I367F^ protein localises to the nucleus. Male *Fto^I367F^* mice exhibit a lean phenotype with reduced body weight and fat mass, despite normal physical activity. This appears to result from an increase in whole body metabolism. The body weight of female mice was unaffected. Sex differences are often observed in mouse models of obesity and diabetes and are thought to be due to a protective effect of female sex hormones [31],[32]. Although most human studies have not reported any gender differences, a recent study suggested that the effect of FTO on BMI in a Slavic population is restricted to males and postmenopausal females [33].

### Properties of FTO

We found that *in vitro* wild-type mFTO dimerises, probably via its novel helical *C*-terminal domain, and that it can exist in both monomeric and dimeric forms. Substitution of isoleucine-367 for phenylalanine resulted in insoluble protein when expressed in *E. coli* and a substantially reduced level of expression in mammalian cells. However alanine substitution at residue 367 resulted in soluble protein. mFTO^I367A^ has a similar secondary structure to mFTO, although the fraction of dimeric protein was significantly reduced and its catalytic activity was decreased.

Our results suggest the mFTO^I367F^ allele may exert a heterozygous hypomorphic effect. Heterozygous *Fto* knockout mice resemble wild-type mice, indicating the presumed FTO reduction is not sufficient to elicit a phenotype (haploinsufficiency). However, because heterozygous and homozygous *Fto^I367F^* mice exhibited a similar phenotype, it appears that an mFTO^I367F^ protein can exert a dominant negative effect on mFTO function, possibly by disrupting the wild-type mFTO subunit by formation of a heterodimeric protein complex (i.e. an antimorphic allele).

ER stress genes were among those genes whose expression was increased in the liver of 16-week old homozygous *Fto^I367F^* mice. It is possible these gene changes are secondary to impaired folding of FTO^I367F^, as suggested by the fact that the recombinant protein aggregated in solution. However, qRT-PCR analysis did not reach statistical significance suggesting FTO^I367F^ may only be initiating a mild unfolded protein response.

### Mechanism of Action of FTO

Most sick animals will tend to lose weight. However, the fact that food intake and physical activity of *Fto^I367F^* mice are normal indicates their lean phenotype is unlikely to be a consequence of illness or general malaise. In contrast, the I367F mutation produced a marked increase in whole body metabolism, with both O_2_ consumption and CO_2_ production being stimulated. This argues that the changes in fat mass are produced by differences in metabolism. Interestingly, RER increased from 0.87 to 0.9 during the dark period (when the mice are active). This is consistent with a switch from fat metabolism (RER = 0.7) towards protein (RER = 0.9) and/or carbohydrate (RER = 1.0) metabolism, which most likely reflects the fact that mutant animals have smaller fat depots.

We found some evidence for alterations in catecholamines and sympathetic nervous system activity that are consistent with an increased metabolic rate. The increased glucagon level of Fto^I367F^ mice further supports an increased sympathetic tone (as this stimulates glucagon secretion).

On either a standard or a high-fat diet, *Fto^I367F^* mice exhibited reduced fat mass, unchanged food intake, and increased serum leptin, glucose, glucagon and lipid serum levels compared with wild-type mice. However the lean mass of *Fto^I367F^* mice was greater than that of wild-type on a high-fat diet, whereas no differences were observed on a normal diet. Consequently, overall weight was not significantly different between *Fto^I367F^* and wild-type mice on a high fat diet. It is striking that that despite the reduction in protein and carbohydrate, the lean mass of *Fto^I367F^* mice increases on the high-fat diet. All mice on the high-fat diet have a higher fat mass than on a standard diet, although the *Fto^I367F^* mutation affords some protection against increased adiposity.

Finally, we did not observe any differences in metabolic rate between genotypes given a high-fat diet, suggesting that the increased fat metabolism (particularly in wild-type mice) masks the differences observed on a standard diet. The RERs on the high-fat diet were all reduced, as expected due to the relatively higher fat metabolism. However, the active (night) phase RER was significantly higher in *Fto^I367F^* than wild-type mice fed a high-fat (or standard) diet, indicating a switch in substrate use towards carbohydrate.

How the *Fto^I367F^* mutation enhances metabolism requires further study. Nevertheless, it is interesting that other Fe(II) and 2OG oxygenases are involved in fat metabolism [34]. Given that the amount of adipose tissue is significantly reduced in *Fto^I367F^* mice, the increased expression of genes involved in lipid metabolism pathways can be interpreted to suggest that fatty acid catabolism dominates in WAT and is the primary cause of reduced WAT levels, and that changes in levels of anabolic genes are a secondary effect. Overall, enhanced fat metabolism in WAT may not contribute to RER as much as in wildtype animals because of the reduced fat mass in *Fto^I367F^* mice.

Gene expression analysis showed a slight increase in carbohydrate metabolic genes in muscle of *Fto^I367F^* mice. This might contribute to the phenotype of these mice as they exhibit a higher metabolic rate and higher RER. It is also possible that these changes are secondary to changes in substrate supply to muscle, produced by changes in nutrient storage in WAT and liver. The elevation of fatty acid synthase (*Fasn*) levels suggests that fat synthesis could be enhanced in muscle. However, there was no obvious increase in fat storage in this tissue so this change may be a secondary consequence of a decreased supply of fatty acids to muscle resulting from the lean phenotype.

A reduction in expression of genes involved in the inflammatory response is consistent with the lean phenotype of the *Fto^I367F^* mice and may reflect concomitant reduction of macrophages residing in WAT. Previous reports have shown inflammation has an important role in obesity and that a lean phenotype results in decreased expression of inflammatory markers [27],[28],[29].

We found a significant reduction in *Npy* expression in *Fto^I367F^* mice in the free-fed state (compared to controls). However, we did not observe any significant effect on food intake. Knockout of FTO had no effect on fed levels of *Npy* expression but produced a small reduction in fasting *Npy* and *Pomc* levels [24].

### Does Loss or Gain of FTO Function Cause Obesity?

Our data suggest that the I367F mutation leads to a reduction in FTO activity. The mutant protein is expressed at lower levels than wild-type in *E. coli*, mammalian cell lines, and in tissues isolated from mutant mice. This reduction in FTO protein levels is expected to result in a decrease in total FTO activity. Furthermore, although the activity of mFTO^I367F^ could not be measured that of mFTO^1367A^ was reduced.

The *Fto^I367F^* mouse shares several features with a mouse in which the *Fto* gene has been knocked out [24], which provides additional support for the idea that the *Fto^I367F^* mutation causes a (partial) loss of function. Thus, in the homozygous state, both types of mice weigh less than wild-type, have a reduced fat mass, exhibit an increased metabolic rate and increased sympathetic nervous system activity and have a stronger phenotype in male than female mice.

Collectively, these data suggest that FTO is involved in fat accumulation and that reduction in the activity of its gene product accounts for the lean phenotype of our mutant mice.

### Differences between *Fto^I367F^* and *Fto* Knockout Mice

There are, however, significant differences between *Fto^I367F^* mice and the *Fto* knockout mouse [24]. This is presumably because the latter results in a null allele, whereas the mFTO^I367F^ protein may retain some functional activity, as suggested by the lowered activity of mFTO^I367A^.

The *Fto* knockout mouse exhibits no significant phenotype in the heterozygous state [24], whereas heterozygous *Fto^I367F^* mice resemble their homozygous littermates. As described above, this most probably results from a dominant negative effect of the I367F mutation. Thus, inhibition of FTO dimerisation may represent a novel strategy for inhibition of FTO activity. We confine our discussion here to a comparison of homozygous animals.

The divergence of weight between knockout and wild-type mice has an early onset being apparent from day 2 after birth, and by 6 weeks of age homozygous mice weigh 30–40% less than controls. In contrast, the weight of homozygous *Fto^I367F^* mice only diverges after 12 weeks and at its maximum (24 weeks) is only 10% less than wild-type. This smaller weight difference is closer to that observed in humans carrying different *FTO* SNPs, where the mean difference between at-risk and low-risk alleles is 3 kg for an average adult body weight of 90 kg - or around 3.4% [1].

*Fto* knockout mice are smaller than wild-type (on average, males are 1.5 cm shorter) [24]. In contrast, *Fto^I367F^* mice showed no difference in body length, which is consistent with the lack of association of human *FTO* SNPs with height [1],[21].

Fat mass was reduced by 60% and 23% in homozygous male and female *Fto* knockout mice, respectively. In contrast, we only observed a phenotype in males and the reduction in fat mass was much less, between 16–18%. This is closer to the difference in body fat of 14% found between humans with at-risk and low-risk *FTO* variants [1].

There are several other differences between *Fto^I367F^* and *Fto* knockout mice. In particular, the latter showed a postnatal increase in mortality suggesting they are less healthy. They also exhibited relative hyperphagia and decreased spontaneous locomotor activity, which we did not detect in *Fto^I367F^* mice.

These data reveal that the phenotype of *Fto^I367F^* mice more closely resembles that of human *FTO* variants than the knockout mice, and suggests that the low-risk allele does not result in a total knockout of FTO function in humans. The *Fto^I367F^* mouse provides a more physiologically relevant model for studying the effects of human *FTO* variants than a complete gene knockout, where the phenotype is more extreme.

### Relation to Human FTO

Human studies suggest that *FTO* SNPs are not associated with differences in physical activity, as found for *Fto^I367F^* mice [20]. There is strong evidence for increased food intake in humans with the ‘at-risk allele’. This was not observed in *Fto^I367F^* mice although homozygotes showed a trend to towards reduced food intake on both standard and high-fat diets. Unlike humans with *FTO* gene variants, we observed no differences in homozygous and heterozygous mice. However, this is not unexpected as we have shown that our mutation affects FTO dimerisation, which may lead to dominant negative effects: it is unlikely this occurs with an intronic SNP, which presumably exerts a gene dosage effect on protein levels.

### Conclusions

Our data provide functional evidence that *FTO* is a causal gene underlying the association of SNPs within intron 1 of *FTO* with obesity. They further suggest that the ‘at-risk’ allele may lead to enhanced FTO function - or conversely that the low risk allele may lead to reduced FTO function.

The *Fto^I367F^* model shows that mutations in this gene are consistent with viable metabolic effects and provides a physiologically more relevant model for further study than does the knockout mutation where the phenotype is more extreme.

Our results cannot exclude the possibility that the intronic SNP found in humans may have an additional regulatory role on other genes. Nevertheless, because a missense point mutation is unlikely to affect the expression of adjacent genes, the phenotype of our mice provides strong support for the idea that FTO function does regulate body weight and further reinforces the evidence from the mouse knockout. By showing that FTO affects whole body metabolism, they provide the basis for further studies designed to elucidate the molecular mechanism of how FTO influences body weight. The observation that impaired FTO function results in a lean phenotype also suggests that inhibition of FTO may be of therapeutic interest in relation to morbid obesity.

## Materials and Methods

### ENU Mutation in Exon 6

The Harwell ENU-DNA archive (http://www.har.mrc.ac.uk/services/dna_archive/) was screened by denaturing high performance liquid chromatography (dHPLC) using a Transgenomic WAVE system [25],[35]. Coding sequence from the 9 *Fto* exons, including flanking (approximately 70 bp) splice sites, was tested in DNA derived from 6,624 mutagenised animals giving a total of approximately 10.29 Mbp screened (see Text S1 for primer sequences). Six ENU induced point mutations (2 missense, 3 intronic (non-splice site), 1 silent) were found. We selected an exon 6 missense mutation (I367F) for further work. *Fto* exon 6 was amplified with the following primers: FTOex6F 5′ ATGTACAGCTGGAAGAGTGC-3′ and FTOex6R 5′-TCCCCTCTGACTAGGATCTC-3′ using Ampli Taq Gold (Applied Biosystems). The mouse (C57BL/6J×C3H/HeH F1) was rederived by *in vitro* fertilization from frozen sperm using C3H/HeH eggs. Progeny were backcrossed for two generations to C3H/HeH and intercrossed to produce mice heterozygous and homozygous for the mutation.

### Sequence Analysis

PCR amplification products were purified using a QIAquick PCR purification kit (Qiagen). Sequencing reactions were performed by GATC Biotech (Jakob-Stadler-Platz 778467 Konstanz, Germany) and analyzed using Vector NTI advance 10-ContigExpress (Invitrogen).

### Sequence Alignment

FTO genomic, transcript and peptide sequences were exported from Ensembl (http://www.ensembl.org/index.html). Sequence alignment was performed using NTI advance 10-AlignX software (Invitrogen).

### Mutant Construct Production

cDNA encoding hFTO was synthesised by Geneart and cloned into the pET-28a(+) vector to generate a His-tagged fusion protein. The hFTO construct used was produced by deletion of the thrombin cleavage region separating the His-tag from the protein sequence, using the QuikChange Site-Directed Mutagenesis Kit (Stratagene), and confirmed by DNA sequencing. This construct was used to prepare the hFTO 332–505 truncation (ChFTO). The mFTO 329–502 truncation (CmFTO), *C*-terminal truncations mFTO 1–408, mFTO 1–387 and mFTO 1–329, and point mutant I367F mFTO and I367A mFTO constructs were prepared in a similar manner from the wild-type mFTO construct. Further details are provided in the Text S1.

### Protein Expression and Purification

Proteins were prepared as previously reported for full length mFTO [8].

### Analytical Gel Filtration Analyses

Protein (250 µg) was loaded onto a Tricorn 10/300 GL column packed with Superdex 75 (CmFTO and CmFTO^I367A^) or Superdex 200 (mFTO and mFTO^I367A^). Chromatographic separation was achieved using one column volume of running buffer, containing 50 mM NaCl, 1 mM DTT, 50 mM Tris pH 7.5. The UV trace of the eluted volume was monitored. Protein standards used to create calibration curves were analysed by the same method. For CmFTO the masses obtained were 23.2 kDa and 38.2 kDa, consistent with the presence of monomeric and dimeric species (calculated masses of 21.6 and 43 kDa, respectively). CmFTO^I367A^ elutes at a volume corresponding to 27.8 kDa.

### Circular Dichroism Analyses

Protein (0.25 mg/ml) samples were analysed in 50 mM KCl, 20 mM K_2_PO_4_, pH 7.0, on an Applied Photophysics Chirascan machine, with 1 mm pathlength quartz cuvettes. Measurements were taken at 0.5 nm intervals between 180 and 260 nm at 4°C. Spectral fitting to determine relative contributions of secondary structure elements was performed using a CDNN deconvolution program (Chirascan).

### Non–Denaturing Mass Spectrometric Analyses

Protein samples were exchanged into 15 mM ammonium acetate, pH 7.5. ESI-MS was carried out on a Waters Micromass Q-ToF spectrometer and an Advion Biosciences NanoMate HD Robot chip-based nano-electrospray device. 10 µM protein samples were sprayed from 15 mM NH_4_OAc (pH 7.5) at a chip nozzle voltage of 1.66 kV and cone voltage of 80 V. Samples were also run on a Waters Synapt HDMS, Advion Biosciences NanoMate HD Robot chip-based nano-electrospray device. 15 µM protein samples were sprayed from 15 mM NH_4_OAc (pH 7.5) at a chip nozzle voltage of 1.7–1.8 kV, a gas pressure of 0.25 psi and cone voltage of 80 V.

### 2OG Decarboxylation Assays

Decarboxylation assays of [1-^14^C]-labelled 2OG were carried out as reported [36], and incubated (37°C for 10 minutes). Assays were carried out with an 18mer methylated oligonucleotide substrate of sequence 5′-GCXAGGTCCCGTAGTGCG-3′, where X is 3-methylthymine, which was synthesised by ATDBio Ltd. (University of Southampton, UK). Assay mixes contained 4 µM enzyme, 100 µM substrate, 4 mM ascorbic acid, 300 µM 2OG (4% [1-^14^C]-2OG), 0.3 mg/ml catalase, 1 mM DTT and 25 µM FeSO_4_.7H_2_O, made to 100 µl with 50 mM Tris pH 7.5.

### Constructs for Fluorescent FTO Fusion Proteins

An *N*-Terminally labelled YFP-Fto fusion construct was generated as described [8]. The I367F variant was created from the wild-type construct using the QuikChange Site-Directed Mutagenesis Kit (Stratagene) and confirmed by DNA sequencing. The primers used were (bold codons indicate the site of mutation): mFTOI367F fwd: 5′- AAA CAA GGA GAG GAA **TTC** CAT AAT GAG GTG GAG -3′mFTOI367F rev: 5′- CTC CAC CTC ATT ATG **GAA** TTC CTC TCC TTG TTT-3′. Cell culture and confocal microscopy are described in Text S1.

The HA-Fto chimera was generated by fusing an HA coding sequence to the *N*-terminus of FTO by PCR. The amplified PCR product was cloned into the pGEM-T Easy vector (Promega), sequenced, and then recloned into the pcDNA3 vector (Invitrogen). The I367F variant was created from the wildtype construct using the QuikChange Site-Directed Mutagenesis Kit (Stratagene).

### Protein Extraction and Immunoblotting

Western blots were performed on 40 µg of total proteins using a rabbit anti recombinant mFTO antibody. Detection was performed using ultrasensitive horseradish Enhanced Chemiluminescence Plus (ECL plus; Amersham). Cos-7 cells expressing HA-FTO or HA-FTOI367F, were lysed with RIPA buffer (containing 1% NP40, 0.5% DOC and 0.1% SDA detergents) and proteins separated by SDS-PAGE. Blots were probed with an anti-HA antibody (Roche) and with an antibody to the TATA-binding protein (Abcam) as a loading control for nuclear proteins.

### Animal Experiments

All animal studies were carried out using guidelines issued by the Medical Research Council in 'Responsibility in the Use of Animals for Medical Research’ (July 1993). Mice were kept in accordance with UK Home Office welfare guidelines and project license restrictions under controlled light (12 hr light and 12 hr dark cycle, dark 7 pm-7 am), temperature (21±2°C) and humidity (55%±10%) conditions. They had free access to water (25 ppm chlorine) and were fed *ad libitum* on a commercial diet (SDS Rat and Mouse No.3 Breeding diet (RM3)) containing 11.5 kcal% fat, 23.93 kcal% protein and 61.57 kcal% carbohydrate. Alternatively, mice were maintained on a high-fat diet (D12451, Research Diets, New Brunswick, NJ, USA) containing 45 kcal% fat, 20 kcal% protein and 35 kcal% carbohydrate. Phenotyping tests were performed according to EMPReSS (European Phenotyping Resource for Standardised Screens from EUMORPHIA) standardized protocols as described at (http://empress.har.mrc.ac.uk).

### Body Mass and Composition

Body mass was measured each week on scales calibrated to 0.01 g. Analysis of body composition was performed by DEXA using the Lunar PIXImus Mouse Densitometer (Wipro GE Healthcare, Madison, WI).

### Measurement of Food Consumption, Blood, Serum, and Urinary Parameters

At 10 and 20 weeks of age mice were placed in metabolic Techniplast cages with free access to water and food. Food consumption was measured by weighing. Urine was collected after 24 hours and urinary catecholamines measured using a 3-CAT Epinephrine, Norepinephrine, Dopamine ELISA (Demeditec) according to the manufacturer's instructions. Creatinine was used to standardize between urine samples. Plasma leptin insulin, adiponectin and glucagon levels were measured using a mouse endocrine Lincoplex kit (Linco research, Missouri, USA) and a Bio-Plex 200 system (Bio-Rad, Hemel Hempstead, UK), according to the manufacturer's instructions. At 24 weeks of age, fasted mice were anaesthetized, killed by exsanguination and blood collected by cardiac puncture. Plasma concentrations of glucose, triglycerides, total cholesterol, HDL cholesterol and LDL cholesterol and urine creatinine were measured on an AU400 (Olympus UK), as described [37].

### Metabolic Rate and Activity Measurements

Metabolic rate was measured at 18 weeks of age using indirect calorimetry (Oxymax; Columbus Instruments) to determine oxygen consumption, carbon dioxide production, respiratory exchange ratio (RER) and heat production. Physical activity was assessed using a Threshold load cell system (Med Associates, VT, USA) over 22 hours and analysed with Threshold Summary, Version 3.0 for upper and lower threshold crossings.

### Gene Expression Profiling

Total ribonucleic acid (RNA) from WAT, skeletal muscle and liver was labelled and hybridised to Affymetrix Mouse GeneST arrays. Arrays were analysed using GeneSpring GX10, GenMAPP and Ingenuity pathway analysis tools. All data is MIAME compliant and has been submitted to ArrayExpress under accession number E-MEXP-2201. Further details of the microarray methodology and SYBR-Green/Taqman (Table S5) PCR assays are described in the Text S1.

### Statistics

Statistical analyses were performed with a Student's t-test for independent samples. Correlation coefficients were obtained by the Pearson method using GraphPad Prism version 5.00 for Windows, GraphPad Software, San Diego California USA, www.graphpad.com. Data are expressed as mean±SEM, and P<0.05 was considered as statistically significant. For microarray studies, differentially expressed genes were identified (after RMA normalization) using an unpaired t-test with a cut-off of P≤0.05. A≥1.5-fold change difference between wildtype and *Fto^I367F^* mice was taken to indicate expression changes between genotypes.

Additional detail on materials and methods is given in Text S1.

## Supporting Information

Figure S1 Non-denaturing mass spectrometric analysis of CmFTO. Peak A corresponds to the predicted mass of the *C*-terminal domain of mFTO, CmFTO (21522 Da). Peak B likely results from α-N-gluconylation of the *N*-terminal His-tag of CmFTO, creating a +178 Da adduct (see Figure S2).
(0.58 MB TIF)

Figure S2 Scheme showing the likely modification of the *N*-terminal His-tag of the CmFTO protein by α-N-gluconylation of an *N*-terminal amino group, creating a +178 Da adduct [3].
(0.46 MB TIF)

Figure S3 Non-denaturing electrospray ionisation mass spectrometry analyses carried out on a Waters Synapt™ HDMS™, showing the oligomeric composition of (i) I367A mFTO, (ii) mFTO and (iii) hFTO.
(0.60 MB TIF)

Figure S4 Secondary structure predictions showing relative proportions of secondary structure elements present in CmFTO (left) and mFTO (right), obtained through spectral fitting of circular dichroism spectra (Figure 2D) using the program CDNN (Chirascan). A greater proportion of α-helix is identified in the C-terminus of mFTO (CmFTO) than for the full length mFTO. Proportions obtained for the analogous proteins for the I367A variants are very similar (data not shown).
(0.31 MB TIF)

Figure S5 Difference circular dichroism spectra, identifying the increased proportion of α-helical content in the *C*-terminal domain relative to the wild-type FTO proteins, and indicating overall structural similarity between the wild type and I367A variants: mFTO-CmFTO (blue), I367A mFTO-I367A CmFTO (red), hFTO-ChFTO (light blue), mFTO-I367A mFTO (green), CmFTO-I367A CmFTO (black).
(0.39 MB TIF)

Figure S6 Circular dichroism spectra showing the secondary structures of hFTO (red), and ChFTO (blue) at 0.25 µM, 4°C.
(0.33 MB TIF)

Figure S7 (A,B) Intraperitoneal glucose tolerance test (A) at 12 weeks following overnight fasting (B) at 16 weeks following overnight fasting. Heterozgyous (I/F, n = 23), homozygous (F/F, n = 18) and wildtype littermate (I/I, n = 22). (C) Circulating overnight fast plasma leptin level at 24 weeks. Heterozgyous (I/F, n = 26), homozygous (F/F, n = 19) and wildtype littermate (I/I, n = 28). (D) Circulating overnight fast plasma leptin level expressed as a function of percentage of body fat at 24 weeks. Heterozgyous (I/F, n = 25), homozygous (F/F, n = 19) and wildtype littermate (I/I, n = 18). (E) Circulating overnight fast plasma insulin level at 24 weeks. Heterozgyous (I/F, n = 26), homozygous (F/F, n = 19) and wildtype littermate (I/I, n = 28). (F) Circulating overnight fast plasma adiponectin level at 24 weeks Heterozgyous (I/F, n = 26), homozygous (F/F, n = 10) and wildtype littermate (I/I, n = 14). (G) Liver triglyceride concentration at 24 weeks. Heterozgyous (I/F, n = 8), homozygous (F/F, n = 7) and wildtype littermate (I/I, n = 8). Data are expressed as mean±SEM, Statistical analysis was carried out using the Student's t-test for differences between *Fto^I367F^* heterozygous or homozygous mice and wild-type littermates.
(0.80 MB TIF)

Figure S8 A-C Body Mass (A) fat mass (B) and lean body mass (C) of 24-week old male heterozygous (n = 20), homozygous (n = 15) and wildtype (n = 9) on high fat diet. (D) Food Intake over 24 hours (10 wk I/I n = 14, I/F n = 22, F/F n = 9, 20 wk I/I n = 12, I/F n = 22, F/F n = 9) on high fat diet. E-G Circulating overnight fasting plasma insulin (E), leptin (F) and glucagon (G) level at 8 and 24 weeks. Heterozgyous (I/F, n = 20), homozygous (F/F, n = 9) and wildtype littermate (I/I, n = 12). (H) Glucose and lipid serum levels were measured after overnight fasting at 24-weeks of age. WT (I/I; n = 11), Heterozygous (I/F; n = 18), Homozygous (F/F; n = 9). I-K Oxygen Consumption (I) and carbon dioxide production (J) and calculated respiratory exchange ratio (RER; K) during the light and dark period for 18-week old males on standard diet (heterozygous (I/F; n = 30) and homozygous (F/F; n = 15) and *Fto^I367F^* mice wild-type littermates (I/I; n = 26) and high fat diet (heterozygous (I/F; n = 23) and homozygous (F/F; n = 9) *Fto^I367F^* mice wild-type littermates (I/I; n = 12). L-M Oxygen Consumption (L) and carbon dioxide production (M) over a 22-hr period for 18- week old male heterozygous (I/F; n = 23) and homozygous (F/F; n = 9) *Fto^I367F^* mice and wild-type littermates (I/I; n = 12) on high fat diet. Data are expressed as mean±SEM, Statistical analysis was carried out using the Student's t-test (* P<0.05, **P<0.01, *** P<0.001) for differences between *Fto^I367F^* heterozygous or homozygous mice and wild-type littermates.
(0.11 MB PDF)

Figure S9 Endoplasmic Reticulum Stress pathway in homozygous *Fto^I367F^* mice liver microarray. Upregulated genes are marked in red and genes showing no change in expression are shown in grey (Downloaded from Ingenuity).
(3.25 MB TIF)

Figure S10 Gene expression in hypothalamus of Agrp, Npy, and Pomc in *Fto^I367F^* expressed relative to GAPDH. Free fed heterozgyous (I/F, n = 6), homozygous (F/F, n = 7) and wildtype littermates (I/I, n = 7). Fasted heterozgyous (I/F, n = 8), homozygous (F/F, n = 8) and wildtype littermate (I/I, n = 8). Data are expressed as mean±SEM, Statistical analysis was carried out using the Student's t-test for differences between *Fto^I367F^* heterozygous or homozygous mice and wild-type littermates. *, P<0.05.
(0.57 MB TIF)

Table S1 Metabolic parameters in female *Fto^I367F^* mice. Values are mean±SEM. Numbers shown in brackets represent n number for each parameter. No statistically significant differences were observed between female wildtype littermates (I/I) and heterozygous (I/F) mice and homozygous (F/F) *Fto^I367F^* mice.
(0.05 MB PDF)

Table S2 Immune genes statistically altered≥1.5 fold in 16 week *Fto^I367F^* white adipose tissue.
(0.07 MB PDF)

Table S3 Fatty acid metabolism genes statistically altered≥1.5 fold in 16 week *Fto^I367F^* white adipose tissue.
(0.02 MB PDF)

Table S4 Metabolism genes statistically altered≥1.5 fold in 16 week *Fto^I367F^* liver.
(0.01 MB PDF)

Table S5 Taqman Gene Expression Assay, probe Assay ID for qRT-PCR.
(0.01 MB PDF)

Text S1 Supplemental data.
(0.06 MB DOC)
