# Supplementary figures and images for: A Mouse Model for the Metabolic Effects of the Human Fat Mass and Obesity Associated FTO Gene
Source: PLoS Genet. 2009 Aug 14;5(8):e1000599. doi: 10.1371/journal.pgen.1000599 (PMC2719869; doi:10.1371/journal.pgen.1000599)

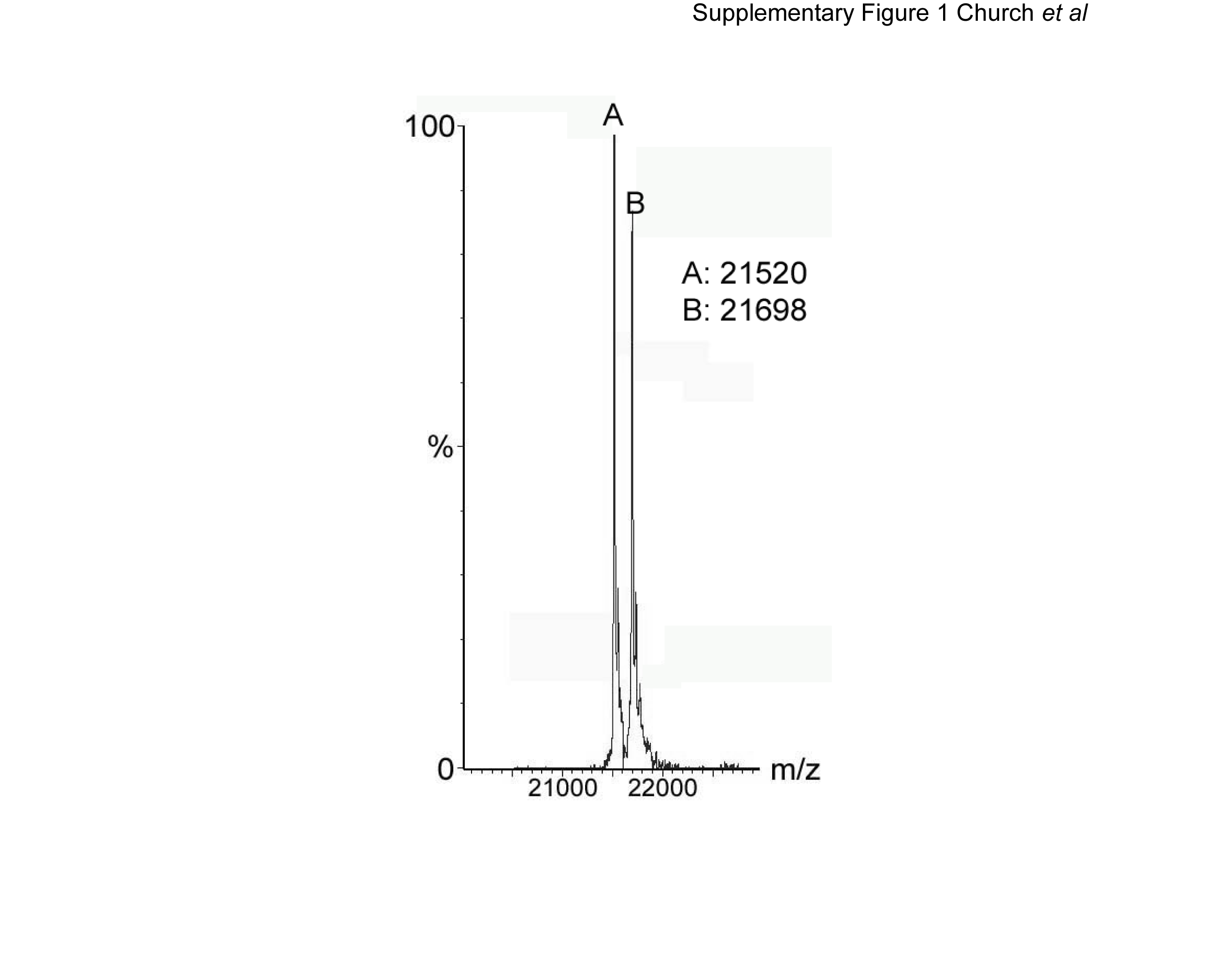

Supplement: Figure S1 — Non-denaturing mass spectrometric analysis of CmFTO. Peak A corresponds to the predicted mass of the C-terminal domain of mFTO, CmFTO (21522 Da). Peak B likely results from α-N-gluconylation of the N-terminal His-tag of CmFTO, creating a +178 Da adduct (see Figure S2). (0.58 MB TIF) [file pgen.1000599.s001.tif]

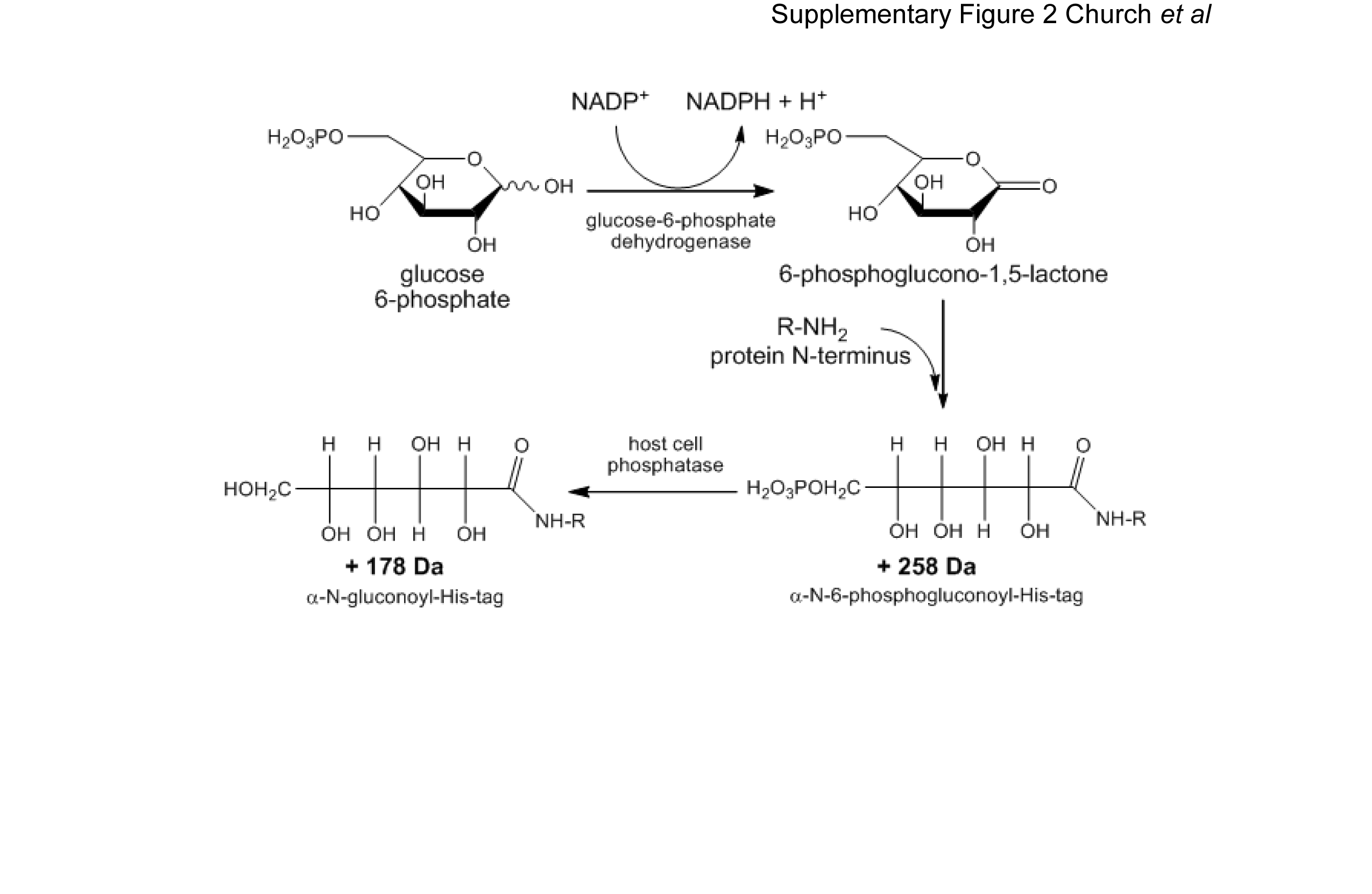

Supplement: Figure S2 — Scheme showing the likely modification of the N-terminal His-tag of the CmFTO protein by α-N-gluconylation of an N-terminal amino group, creating a +178 Da adduct [3]. (0.46 MB TIF) [file pgen.1000599.s002.tif]

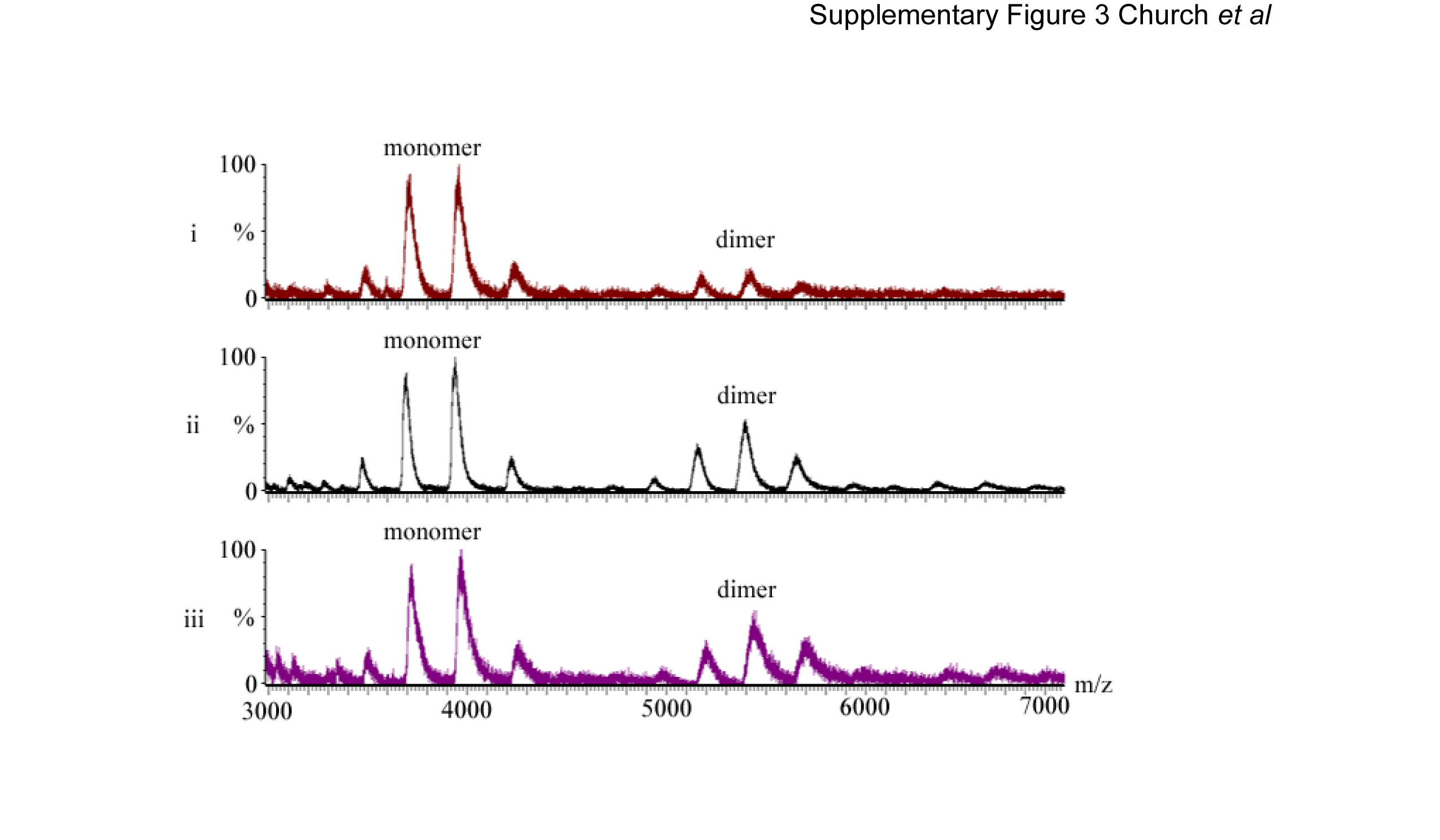

Supplement: Figure S3 — Non-denaturing electrospray ionisation mass spectrometry analyses carried out on a Waters Synapt™ HDMS™, showing the oligomeric composition of (i) I367A mFTO, (ii) mFTO and (iii) hFTO. (0.60 MB TIF) [file pgen.1000599.s003.tif]

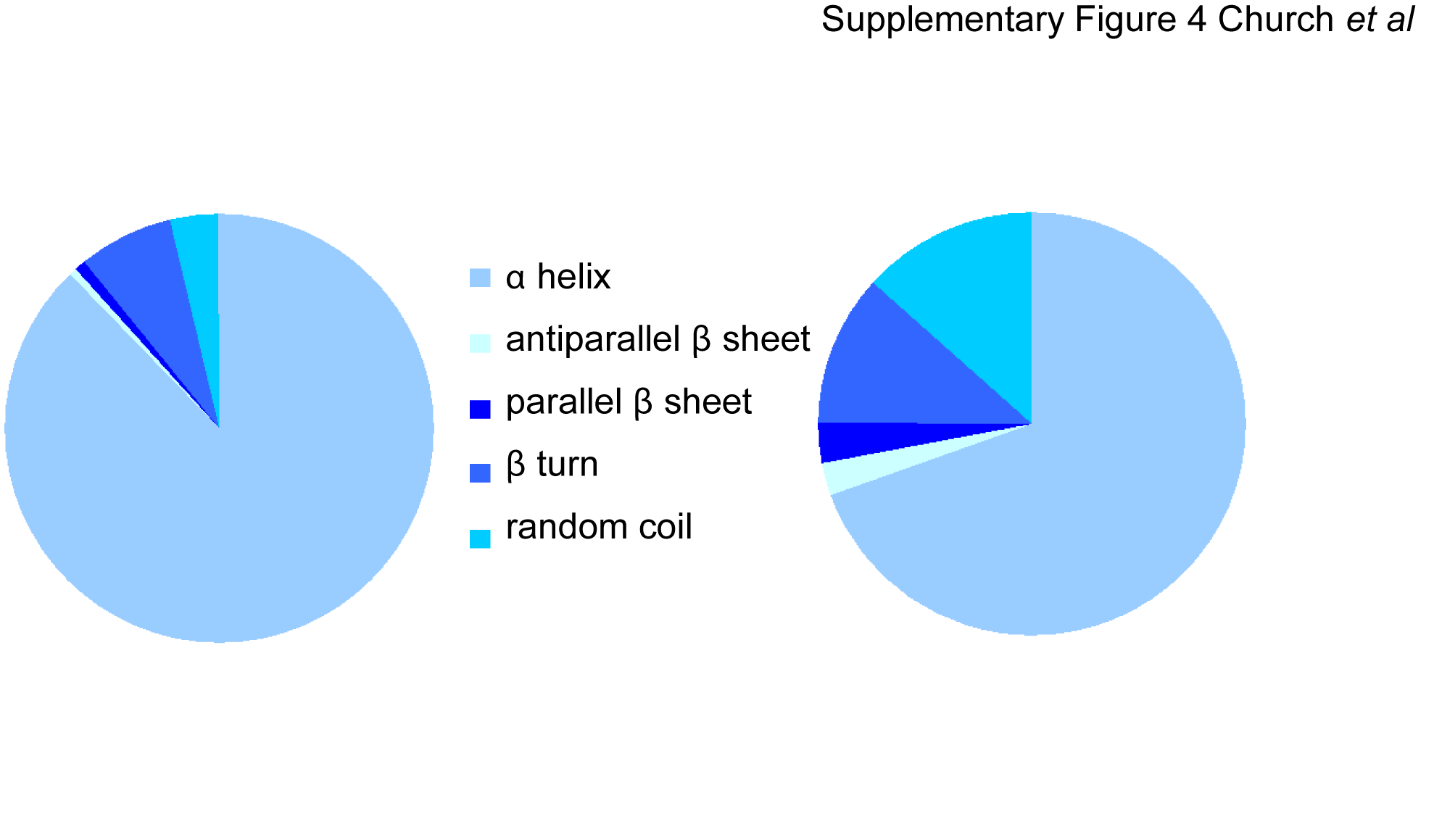

Supplement: Figure S4 — Secondary structure predictions showing relative proportions of secondary structure elements present in CmFTO (left) and mFTO (right), obtained through spectral fitting of circular dichroism spectra (Figure 2D) using the program CDNN (Chirascan). A greater proportion of α-helix is identified in the C-terminus of mFTO (CmFTO) than for the full length mFTO. Proportions obtained for the analogous proteins for the I367A variants are very similar (data not shown). (0.31 MB TIF) [file pgen.1000599.s004.tif]

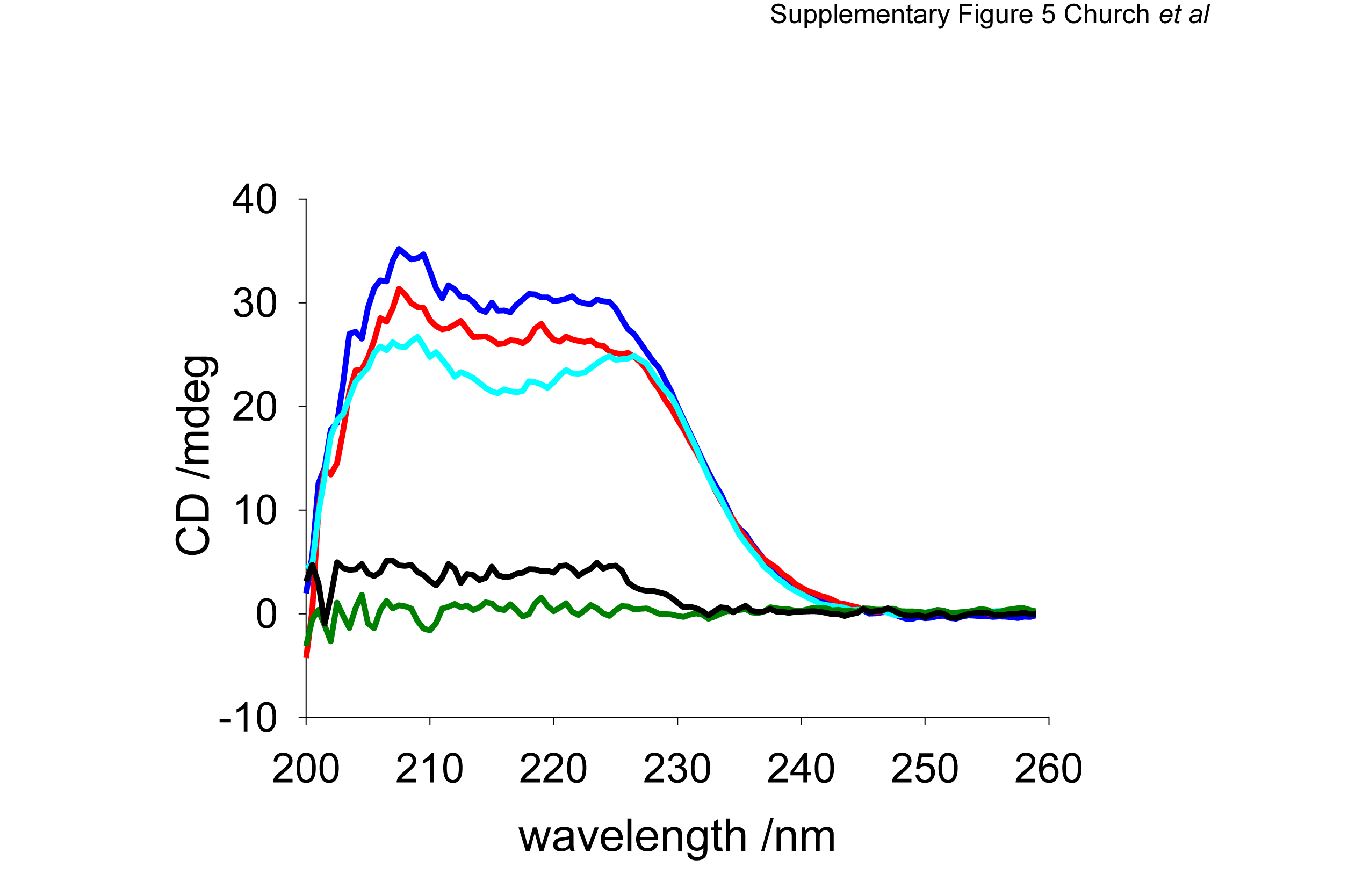

Supplement: Figure S5 — Difference circular dichroism spectra, identifying the increased proportion of α-helical content in the C-terminal domain relative to the wild-type FTO proteins, and indicating overall structural similarity between the wild type and I367A variants: mFTO-CmFTO (blue), I367A mFTO-I367A CmFTO (red), hFTO-ChFTO (light blue), mFTO-I367A mFTO (green), CmFTO-I367A CmFTO (black). (0.39 MB TIF) [file pgen.1000599.s005.tif]

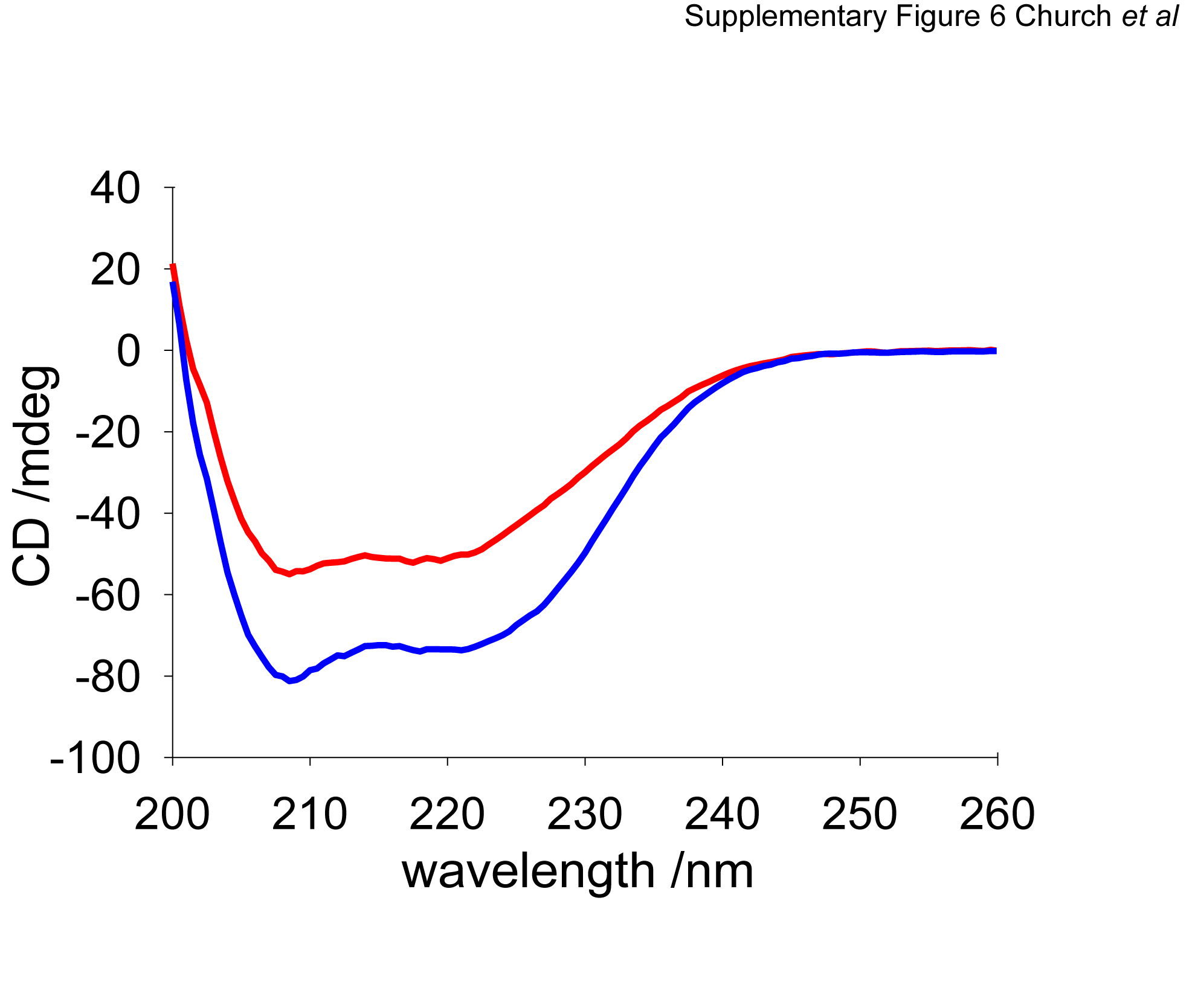

Supplement: Figure S6 — Circular dichroism spectra showing the secondary structures of hFTO (red), and ChFTO (blue) at 0.25 µM, 4°C. (0.33 MB TIF) [file pgen.1000599.s006.tif]

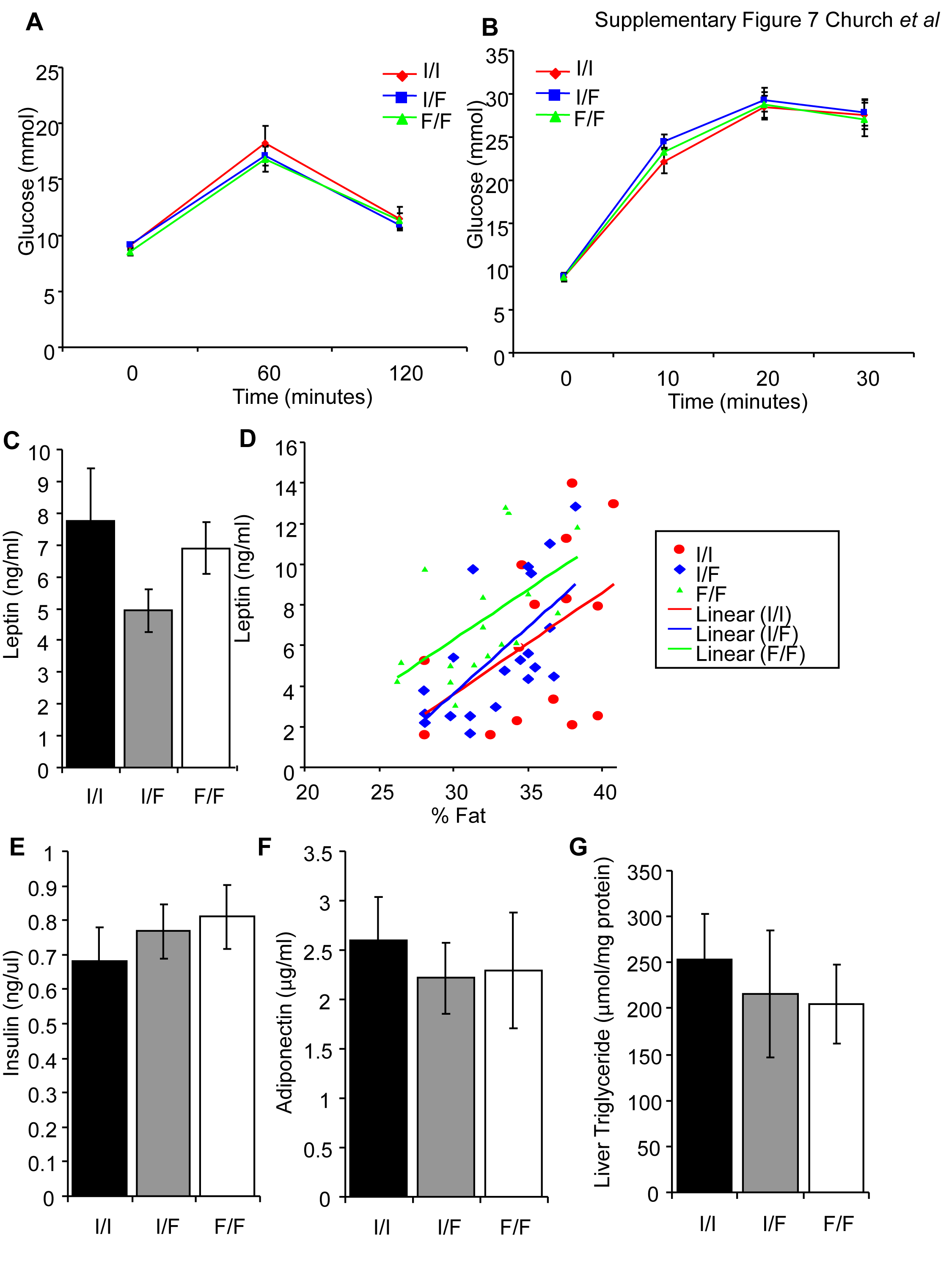

Supplement: Figure S7 — (A,B) Intraperitoneal glucose tolerance test (A) at 12 weeks following overnight fasting (B) at 16 weeks following overnight fasting. Heterozgyous (I/F, n = 23), homozygous (F/F, n = 18) and wildtype littermate (I/I, n = 22). (C) Circulating overnight fast plasma leptin level at 24 weeks. Heterozgyous (I/F, n = 26), homozygous (F/F, n = 19) and wildtype littermate (I/I, n = 28). (D) Circulating overnight fast plasma leptin level expressed as a function of percentage of body fat at 24 weeks. Heterozgyous (I/F, n = 25), homozygous (F/F, n = 19) and wildtype littermate (I/I, n = 18). (E) Circulating overnight fast plasma insulin level at 24 weeks. Heterozgyous (I/F, n = 26), homozygous (F/F, n = 19) and wildtype littermate (I/I, n = 28). (F) Circulating overnight fast plasma adiponectin level at 24 weeks Heterozgyous (I/F, n = 26), homozygous (F/F, n = 10) and wildtype littermate (I/I, n = 14). (G) Liver triglyceride concentration at 24 weeks. Heterozgyous (I/F, n = 8), homozygous (F/F, n = 7) and wildtype littermate (I/I, n = 8). Data are expressed as mean±SEM, Statistical analysis was carried out using the Student's t-test for differences between FtoI367F heterozygous or homozygous mice and wild-type littermates. (0.80 MB TIF) [file pgen.1000599.s007.tif]

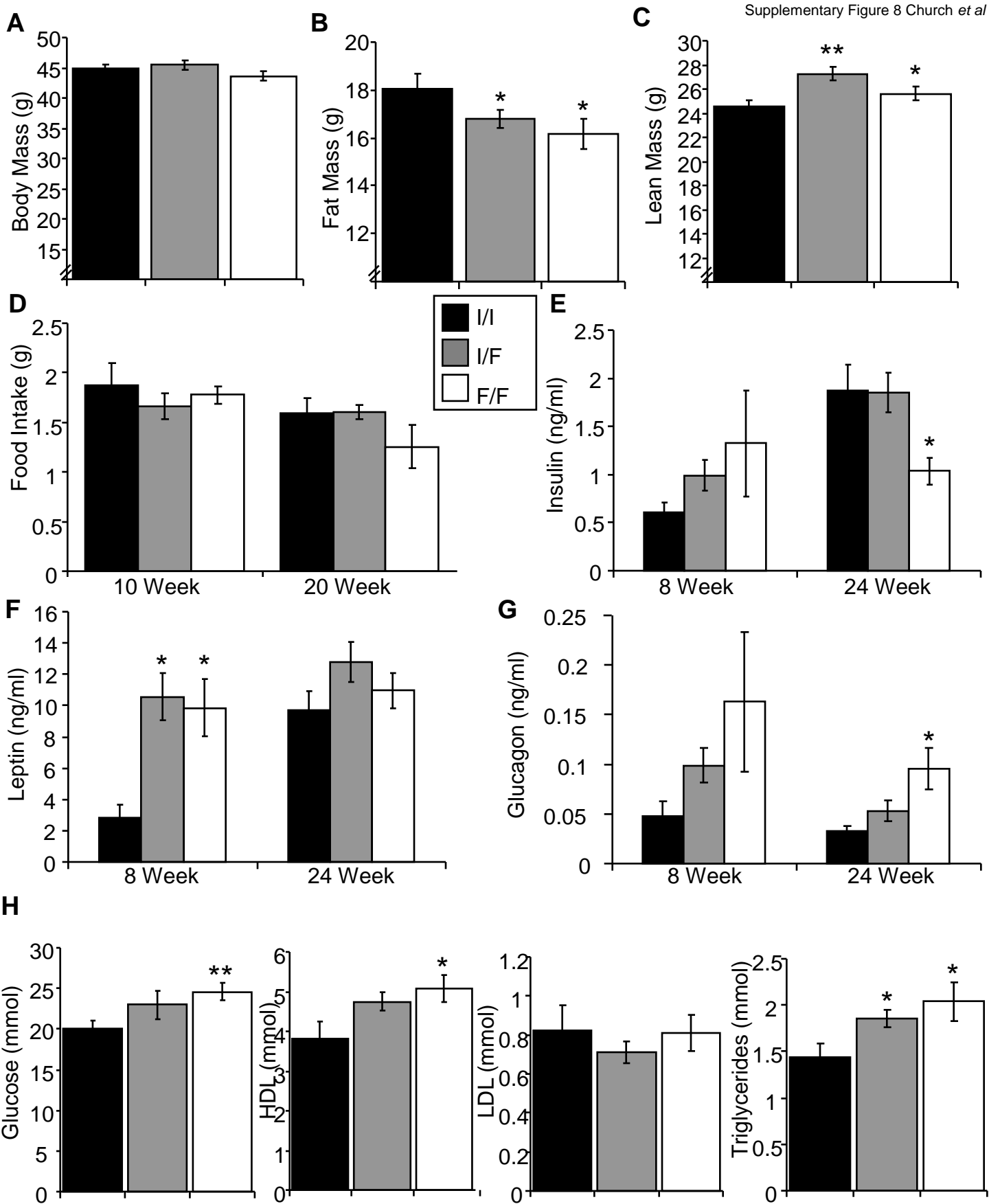

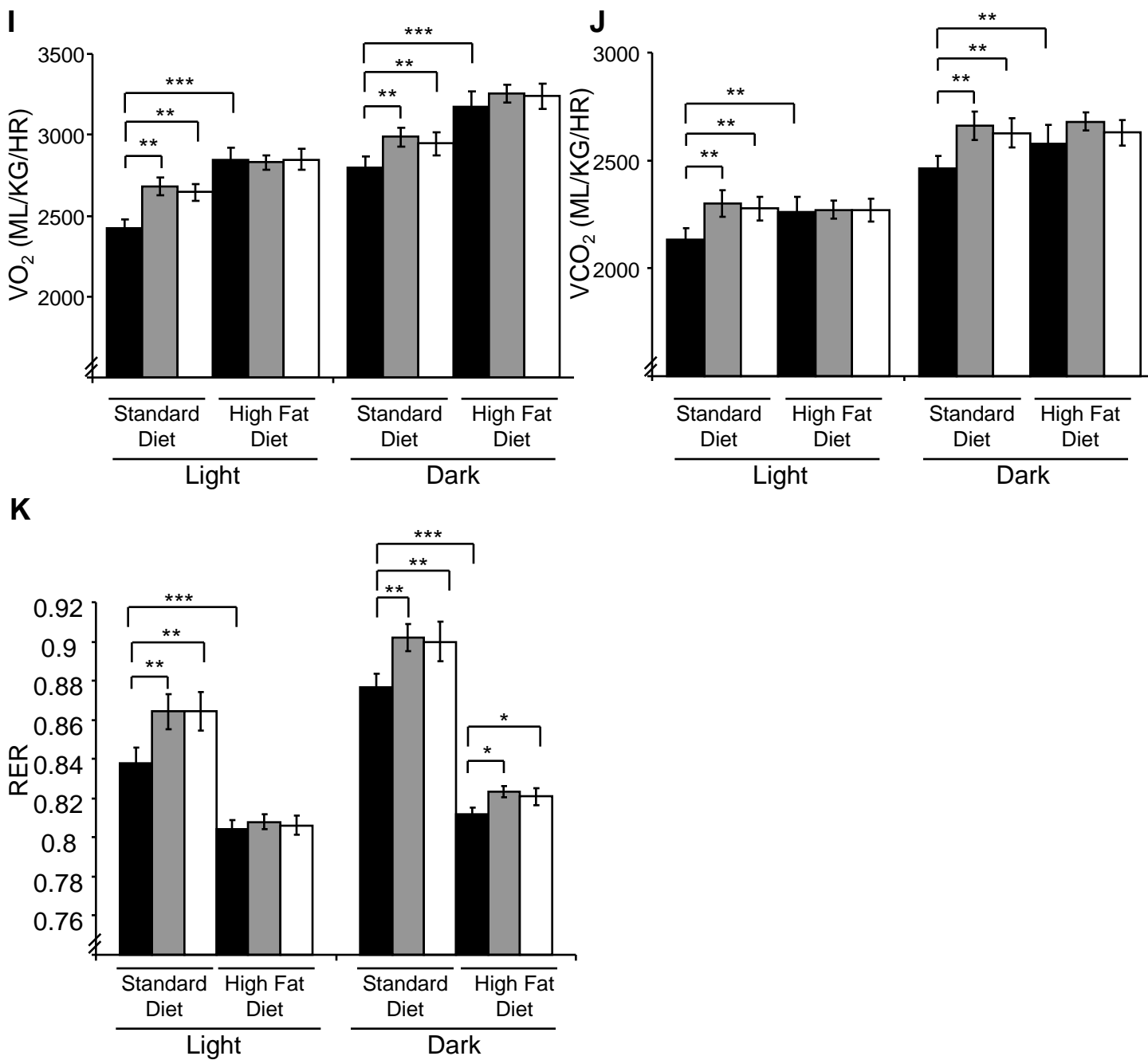

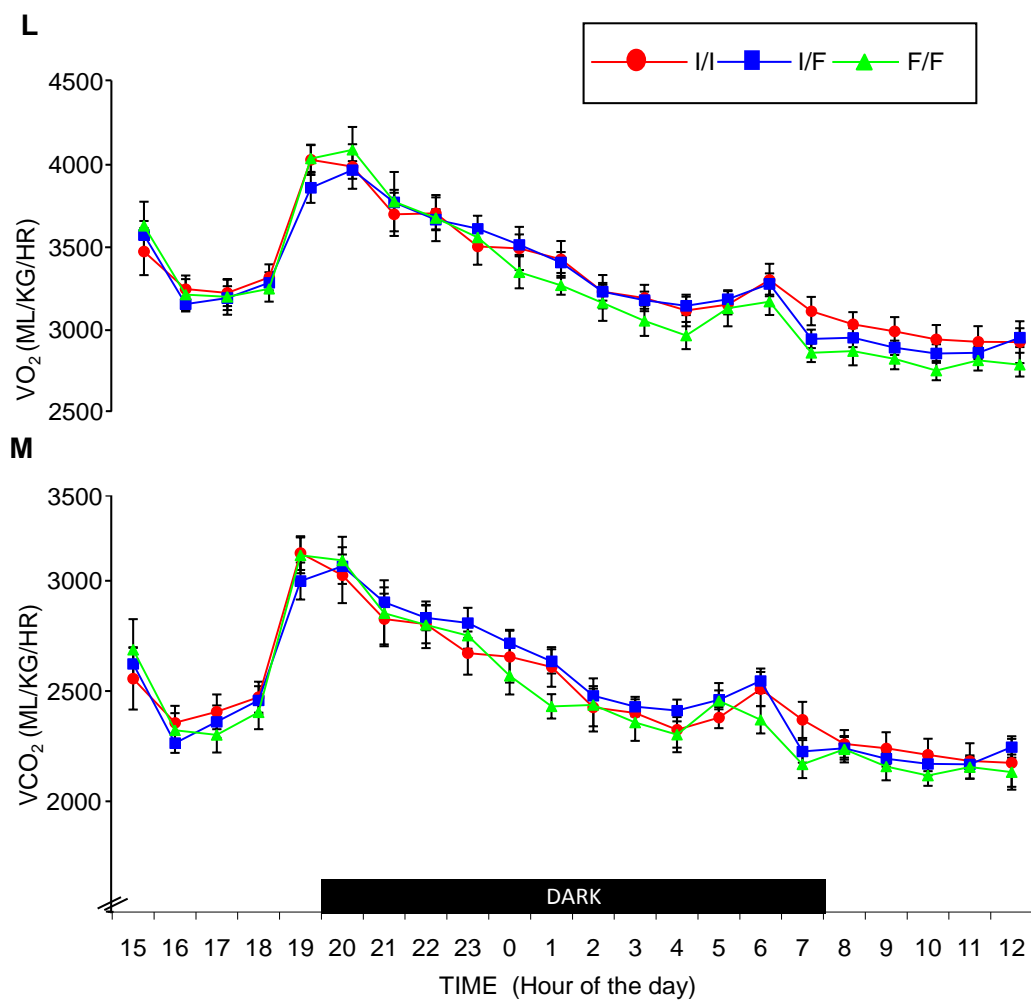

Supplement: Figure S8 — A-C Body Mass (A) fat mass (B) and lean body mass (C) of 24-week old male heterozygous (n = 20), homozygous (n = 15) and wildtype (n = 9) on high fat diet. (D) Food Intake over 24 hours (10 wk I/I n = 14, I/F n = 22, F/F n = 9, 20 wk I/I n = 12, I/F n = 22, F/F n = 9) on high fat diet. E-G Circulating overnight fasting plasma insulin (E), leptin (F) and glucagon (G) level at 8 and 24 weeks. Heterozgyous (I/F, n = 20), homozygous (F/F, n = 9) and wildtype littermate (I/I, n = 12). (H) Glucose and lipid serum levels were measured after overnight fasting at 24-weeks of age. WT (I/I; n = 11), Heterozygous (I/F; n = 18), Homozygous (F/F; n = 9). I-K Oxygen Consumption (I) and carbon dioxide production (J) and calculated respiratory exchange ratio (RER; K) during the light and dark period for 18-week old males on standard diet (heterozygous (I/F; n = 30) and homozygous (F/F; n = 15) and FtoI367F mice wild-type littermates (I/I; n = 26) and high fat diet (heterozygous (I/F; n = 23) and homozygous (F/F; n = 9) FtoI367F mice wild-type littermates (I/I; n = 12). L-M Oxygen Consumption (L) and carbon dioxide production (M) over a 22-hr period for 18- week old male heterozygous (I/F; n = 23) and homozygous (F/F; n = 9) FtoI367F mice and wild-type littermates (I/I; n = 12) on high fat diet. Data are expressed as mean±SEM, Statistical analysis was carried out using the Student's t-test (* P<0.05, **P<0.01, *** P<0.001) for differences between FtoI367F heterozygous or homozygous mice and wild-type littermates. (0.11 MB PDF) [file pgen.1000599.s008.pdf]

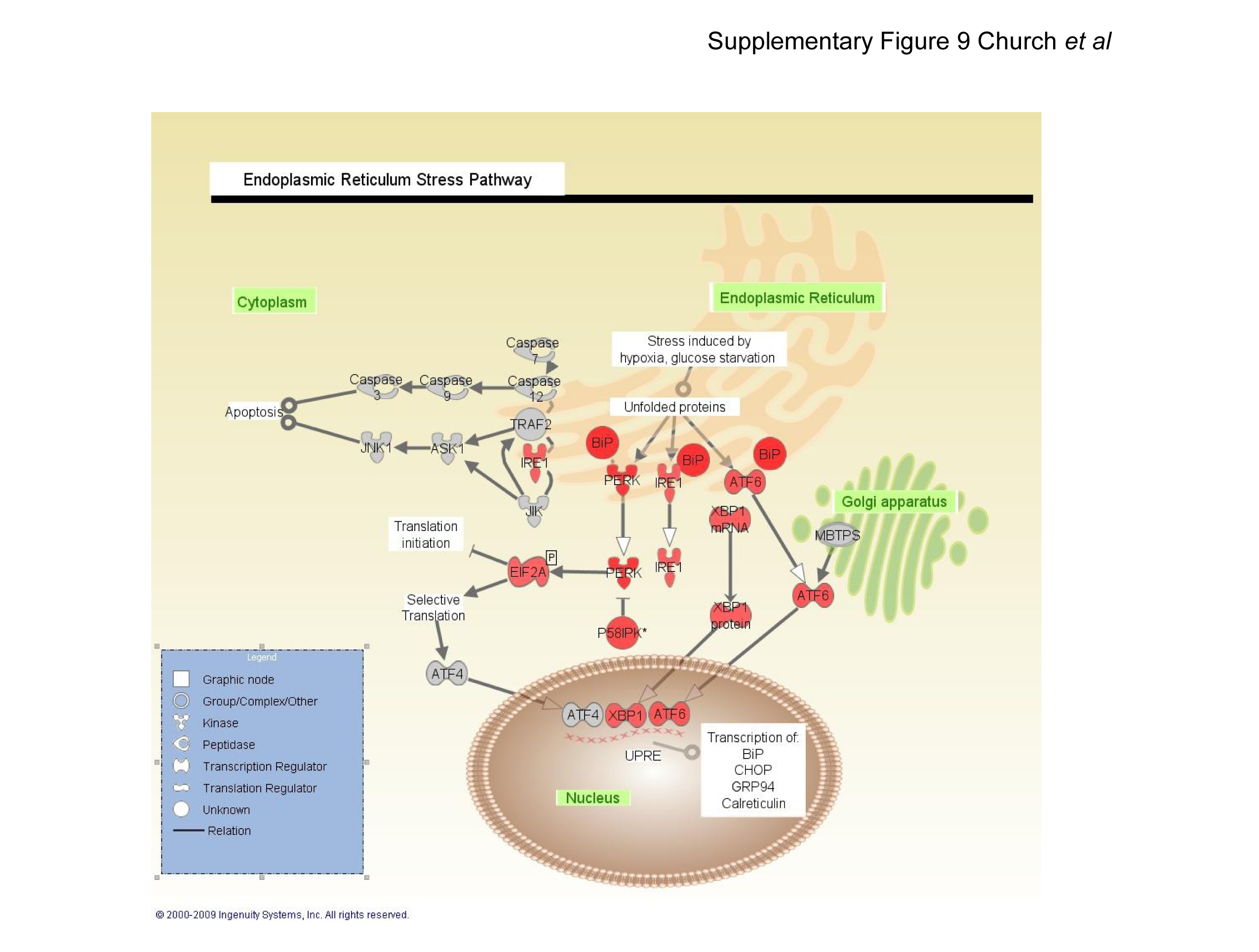

Supplement: Figure S9 — Endoplasmic Reticulum Stress pathway in homozygous FtoI367F mice liver microarray. Upregulated genes are marked in red and genes showing no change in expression are shown in grey (Downloaded from Ingenuity). (3.25 MB TIF) [file pgen.1000599.s009.tif]

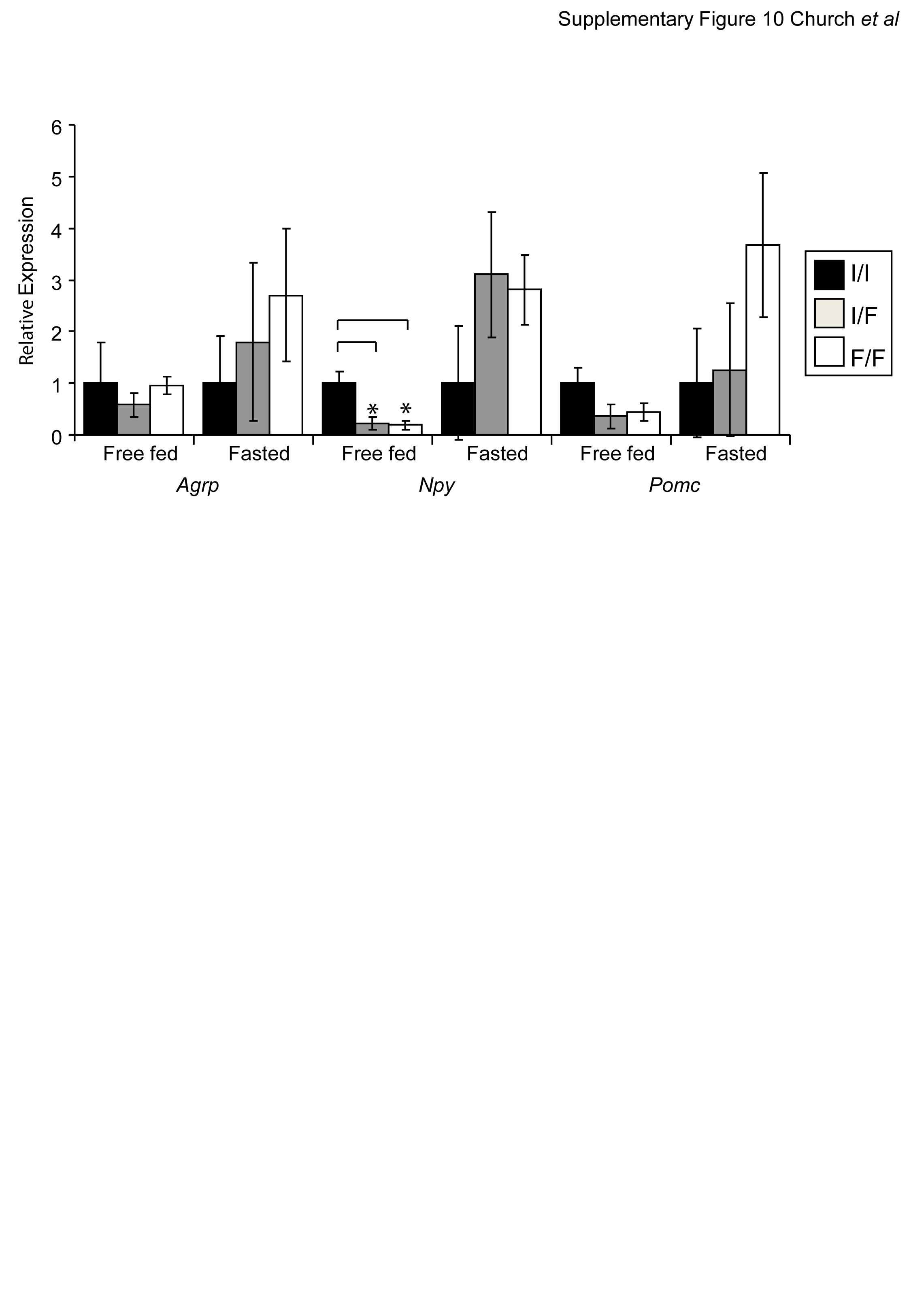

Supplement: Figure S10 — Gene expression in hypothalamus of Agrp, Npy, and Pomc in FtoI367F expressed relative to GAPDH. Free fed heterozgyous (I/F, n = 6), homozygous (F/F, n = 7) and wildtype littermates (I/I, n = 7). Fasted heterozgyous (I/F, n = 8), homozygous (F/F, n = 8) and wildtype littermate (I/I, n = 8). Data are expressed as mean±SEM, Statistical analysis was carried out using the Student's t-test for differences between FtoI367F heterozygous or homozygous mice and wild-type littermates. *, P<0.05. (0.57 MB TIF) [file pgen.1000599.s010.tif]
