# Supplementary material for: A Mouse Model for the Metabolic Effects of the Human Fat Mass and Obesity Associated FTO Gene
Source: PLoS Genet. 2009 Aug 14;5(8):e1000599. doi: 10.1371/journal.pgen.1000599 (PMC2719869; doi:10.1371/journal.pgen.1000599)
Supplement: Table S1 — Metabolic parameters in female FtoI367F mice. Values are mean±SEM. Numbers shown in brackets represent n number for each parameter. No statistically significant differences were observed between female wildtype littermates (I/I) and heterozygous (I/F) mice and homozygous (F/F) FtoI367F mice. (0.05 MB PDF) [file pgen.1000599.s011.pdf]

Parameter	Unit	I/I	I/F	F/F
10 Week Food intake	g	4.38 ± 0.14 (25)	4.39 ± 0.29 (19)	3.99 ± 0.24 (8)
12 Week IPGTT glucose (T=0 minutes) glucose	mmol	7.00 ± 0.30 (20)	7.21 ± 0.36 (16)	8.11 ± 0.38 (7)
12 Week IPGTT glucose (T=60 minutes) glucose	mmol	11.57 ± 0.39 (20)	10.71 ± 0.47 (16)	12.16 ± 1.65 (7)
12 Week IPGTT glucose (T=60 minutes) glucose	mmol	8.57 ± 0.26 (20)	8.09 ± 0.45 (16)	9.64 ± 0.71 (7)
16 Week IPGTT glucose (T=0 minutes) glucose	mmol	8.54 ± 0.97 (12)	8.27 ± 1.34 (9)	8.41 ± 0.71 (5)
16 Week IPGTT glucose (T=10 minutes) glucose	mmol	17.1 ± 1.58 (12)	18.89 ± 2.29 (9)	19.22 ± 3.01 (5)
16 Week IPGTT glucose (T=20 minutes) glucose	mmol	19.41 ± 1.68 (12)	18.95 ± 2.40 (9)	20.01 ± 2.87 (5)
16 Week IPGTT glucose (T=30 minutes) glucose	mmol	16.62 ± 2.22 (12)	15.17 ± 2.89 (9)	16.81 ± 3.11 (5)
24 Week Body Mass	g	36.02 ± 0.97 (20)	34.64 ± 0.72 (19)	36.62 ± 1.94 (9)
24 Week Lean Mass	g	19.85 ± 0.52 (20)	19.84 ± 0.729 (19)	21.47 ± 1.73 (9)
24 Week Fat Mass	g	14.66 ± 0.79 (20)	13.52 ± 0.763 (19)	15.21 ± 1.68 (9)
24 Week Fasting Glucose	mmol	7.69 ± 0.25 (20)	7.84 ± 0.763 (18)	6.47 ± 0.763 (9)
24 Week Fasting Insulin	ng/ml	0.25 ± 0.03 (20)	0.49 ± 0.763 (18)	0.31 ± 0.12 (9)
24 Week Fasting Leptin	ng/ml	0.74 ± 0.14 (20)	0.77 ± 0.10 (18)	0.56 ± 0.10 (9)
24 Week Fasting Glucagon	ng/ml	0.12 ± 0.01 (20)	0.12 ± 0.03 (18)	0.10 ± 0.05 (9)
