# Supplementary material for: A Mouse Model for the Metabolic Effects of the Human Fat Mass and Obesity Associated FTO Gene
Source: PLoS Genet. 2009 Aug 14;5(8):e1000599. doi: 10.1371/journal.pgen.1000599 (PMC2719869; doi:10.1371/journal.pgen.1000599)
Supplement: Table S2 — Immune genes statistically altered≥1.5 fold in 16 week FtoI367F white adipose tissue. (0.07 MB PDF) [file pgen.1000599.s012.pdf]

Probeset ID	Gene Symbol	Fold change	Refseq ID
10541614	Clec4d	-7.9	NM_010819
10445293	Pla2g7	-6.2	NM_013737
10363070	Gp49a	-4.8	NM_008147
10363082	Lilrb4	-4.7	NM_013532
10601385	Tlr13	-4.5	NM_205820
10364262	Itgb2	-4.3	NM_008404
10392808	4732429D16Rik	-4.2	NM_145437
10598976	Timp1	-4.2	NM_001044384
10392815	AF251705	-3.9	NM_134158
10534303	Lat2	-3.8	NM_020044
10379535	Ccl8	-3.8	NM_021443
10360158	Ly9	-3.6	NM_008534
10578264	Msr1	-3.6	NM_001113326
10579347	Ifi30	-3.6	NM_023065
10534202	Ncf1	-3.6	NM_010876
10547657	C3ar1	-3.5	NM_009779
10548375	Clec7a	-3.4	NM_020008
10392796	Cd300lb	-3.3	NM_199221
10473809	Sfpi1	-3.3	NM_011355
10541605	Clec4n	-3.3	NM_020001
10542164	Clec12a	-3.0	NM_177686
10508734	Ptafr	-2.9	NM_001081211
10557862	Itgam	-2.9	NM_001082960
10416437	Lcp1	-2.8	NM_008879
10430372	Rac2	-2.8	NM_009008
10385118	Dock2	-2.7	NM_033374
10360070	Fcer1g	-2.7	NM_010185
10446253	Vav1	-2.7	NM_011691
10389222	Ccl6	-2.6	NM_009139
10582162	Cotl1	-2.6	NM_028071
10573348	Cacna1a	-2.6	NM_007578
10382438	Cd300a	-2.6	NM_170758
10392834	RP23-331L12.7	-2.5	NM_001101657
10500335	Fcgr1	-2.5	NM_010186
10518300	Tnfrsf1b	-2.5	NM_011610
10389231	Ccl3	-2.5	NM_011337
10404606	Ly86	-2.5	NM_010745
10392825	RP23-331L12.7	-2.5	NM_001101657
10560242	C5ar1	-2.5	NM_007577
10372648	Lyz2	-2.5	NM_017372
10607868	Tlr8	-2.5	NM_133212
10360040	Fcgr3	-2.5	NM_010188

10398267	Evl	-2.4	NM_007965
10358224	Ptprc	-2.4	NM_001111316
10489391	Ada	-2.4	NM_007398
10406928	Cd180	-2.4	NM_008533
10532744	Selplg	-2.3	NM_009151
10547769	Ptpn6	-2.3	NM_013545
10441003	Runx1	-2.3	NM_001111023
10458382	Cd14	-2.3	NM_009841
10405216	Syk	-2.3	NM_011518
10348244	Inpp5d	-2.3	NM_010566
10485405	Cd44	-2.2	NM_009851
10598013	Ccr5	-2.1	NM_009917
10590635	Ccr5	-2.1	NM_009917
10453747	Colec12	-2.1	NM_130449
10349051	Tnfrsf11a	-2.1	NM_009399
10375145	Lcp2	-2.1	NM_010696
10389214	Ccl9	-2.0	NM_011338
10411595	Naip2	-2.0	NM_010872
10418410	Prkcd	-2.0	NM_011103
10435565	Hcls1	-2.0	NM_008225
10344966	Ly96	-2.0	NM_016923
10508074	Csf3r	-2.0	NM_007782
10517508	C1qb	-2.0	NM_009777
10598004	Ccr1	-2.0	NM_009912
10573461	Dnase2a	-2.0	NM_010062
10521759	Slit2	-1.9	NM_178804
10576034	Irf8	-1.9	NM_008320
10597461	Cmtm7	-1.9	NM_133978
10518686	Pik3cd	-1.9	NM_008840
10530145	Tlr1	-1.8	NM_030682
10403871	Aoah	-1.8	NM_012054
10544273	Clec5a	-1.8	NM_001038604
10444814	H2-Q2	-1.8	NM_010393
10418506	Stab1	-1.8	NM_138672
10574471	Cmtm3	-1.8	NM_024217
10603860	Cfp	-1.8	NM_008823
10532839	Trpv4	-1.8	NM_022017
10387890	Cxcl16	-1.8	NM_023158
10444821	H2-Q8	-1.8	NM_023124
10601360	Atp7a	-1.8	NM_001109757
10530151	Tlr6	-1.8	NM_011604
10404996	Ninj1	-1.7	NM_013610
10444258	Psmb8	-1.7	NM_010724
10607870	Tlr7	-1.7	NM_133211

10450682	H2-T23	-1.7	NM_010398
10517513	C1qc	-1.7	NM_007574
10517517	C1qa	-1.7	NM_007572
10387568	Tnfsf12	-1.7	NM_001034097
10436841	Il10rb	-1.7	NM_008349
10450675	H2-T24	-1.7	NM_008207
10374333	Ikzf1	-1.7	NM_001025597
10524621	Oasl2	-1.7	NM_011854
10521913	Rbpj	-1.6	NM_009035
10458816	Ticam2	-1.6	NM_173394
10551185	Tgfb1	-1.6	NM_011577
10503098	Lyn	-1.6	NM_001111096
10424400	Myc	-1.6	NM_010849
10567964	Cln3	-1.6	NM_009907
10574456	Cklf	-1.6	NM_001037841
10569102	Irf7	-1.6	NM_016850
10519324	Cdk6	-1.6	NM_009873
10468949	Dclre1c	-1.6	NM_146114
10548286	Klrb1a	-1.6	NM_010737
10371846	Apaf1	-1.5	NM_001042558
10385500	Irgm	-1.5	NM_008326
10356278	Sp110	-1.5	NM_175397
10567825	Lat	-1.5	NM_010689
10597648	Myd88	-1.5	NM_010851
10605357	Gab3	-1.5	NM_181584
10521090	Tacc3	-1.5	NM_001040435
10444244	Tap1	-1.5	NM_013683
10454198	Rnf125	1.5	NM_026301
10593225	Zbtb16	1.6	NM_001033324
10602372	Alas2	1.6	NM_009653
10581650	Chst4	1.7	NM_011998
10511363	Penk1	1.8	NM_001002927
10497079	Ptger3	2.2	NM_011196
10474700	Thbs1	2.3	NM_011580
10593167	Tcea1	3.4	NM_011541
