# Supplementary material for: A Mouse Model for the Metabolic Effects of the Human Fat Mass and Obesity Associated FTO Gene
Source: PLoS Genet. 2009 Aug 14;5(8):e1000599. doi: 10.1371/journal.pgen.1000599 (PMC2719869; doi:10.1371/journal.pgen.1000599)
Supplement: Table S3 — Fatty acid metabolism genes statistically altered≥1.5 fold in 16 week FtoI367F white adipose tissue. (0.02 MB PDF) [file pgen.1000599.s013.pdf]

Probeset ID	Gene Symbol	Fold change	Refseq ID
10558673	Cyp2e1	6.6	NM_021282
10463051	Cyp2c50	3.5	NM_134144
10535704	Cyp3a11	3.4	NM_007818
10379820	Acaca	2.6	NM_133360
10430883	Cyp2d13	2.5	NR_003552
10463043	Cyp2c37	2.4	NM_010001
10393970	Fasn	2.2	NM_007988
10524460	Acacb	2.1	NM_133904
10461979	Aldh1a1	2	NM_013467
10408335	Aldh5a1	1.9	NM_172532
10466624	Aldh1a7	1.9	NM_011921
10477649	Acss2	1.9	NM_019811
10580233	Gcdh	1.9	NM_008097
10372208	Acss3	1.8	NM_198636
10425822	Pnpla3	1.8	NM_054088
10347741	Mogat1	1.7	NM_026713
10453057	Cyp1b1	1.7	NM_009994
10344725	Adhfe1	1.6	NM_175236
10438575	Ehhadh	1.6	NM_023737
10460400	Pcx	1.6	NM_008797
10474836	Ivd	1.6	NM_019826
10565786	Mogat2	1.5	NM_177448
