# Supplementary material for: A Mouse Model for the Metabolic Effects of the Human Fat Mass and Obesity Associated FTO Gene
Source: PLoS Genet. 2009 Aug 14;5(8):e1000599. doi: 10.1371/journal.pgen.1000599 (PMC2719869; doi:10.1371/journal.pgen.1000599)
Supplement: Table S4 — Metabolism genes statistically altered≥1.5 fold in 16 week FtoI367F liver. (0.01 MB PDF) [file pgen.1000599.s014.pdf]

Probeset ID	Gene Symbol	Fold change	Refseq ID
10393970	Fasn	10.2	NM_007988
10605338	G6pdx	3.5	NM_008062
10534883	Irs1	1.6	NM_010571
10543017	Pdk4	1.9	NM_013743
