# Supplementary material for: A Mouse Model for the Metabolic Effects of the Human Fat Mass and Obesity Associated FTO Gene
Source: PLoS Genet. 2009 Aug 14;5(8):e1000599. doi: 10.1371/journal.pgen.1000599 (PMC2719869; doi:10.1371/journal.pgen.1000599)
Supplement: Table S5 — Taqman Gene Expression Assay, probe Assay ID for qRT-PCR. (0.01 MB PDF) [file pgen.1000599.s015.pdf]

Acaca	Mm01304270_m1
Acaa2	Mm00480101_m1
Acacb	Mm01204678_m1
Acsl5	Mm01261084_g1
Acss2	Mm00624282_m1
Adrb1	Mm00431701_s1
Adrb2	Mm02524224_s1
Adrb3	Mm00442669_m1
Comt	Mm00514377_m1
Fasn	Mm00662319_m1
G6pd	Mm00451435_m1
Irs1	Mm00439720_s1
Itgb2	Mm00434523_g1
Lilrb4	Mm00496572_m1
Mogat1	Mm00624192_m1
Mogat2	Mm00503358_m1
Msr1	Mm00446214_m1
Pcx	Mm00500992_m1
Pdk4	Mm01166879_m1
Pla2g7	Mm00479105_m1
Pnpla3	Mm00504420_m1
Ppp1r3c	Mm01204084_m1
Ucp1	Mm01244861_m1
Ucp2	Mm00495907_g1

.
