# Supplementary material for: A Mouse Model for the Metabolic Effects of the Human Fat Mass and Obesity Associated FTO Gene
Source: PLoS Genet. 2009 Aug 14;5(8):e1000599. doi: 10.1371/journal.pgen.1000599 (PMC2719869; doi:10.1371/journal.pgen.1000599)
Supplement: Text S1 — Supplemental data. (0.06 MB DOC) [file pgen.1000599.s016.doc]

**Text S1- Supplemental Data**

**A Mouse Model for the Metabolic Effects of the Human Fat Mass and Obesity Associated *FTO* Gene**

Chris Church, Sheena Lee, Eleanor A L Bagg, James S McTaggart, Robert Deacon, Thomas Gerken, Angela Lee, Lee Moir, Jasmin Mecinović, Mohamed M Quwailid, Christopher J Schofield, Frances M Ashcroft, Roger D Cox.

**Supplemental Experimental Procedures**

**ENU Archive Screen**

Primers used to screen *Fto* genomic sequence from the Harwell ENU-induced mutagenesis archive:

Ftoex1F 5’-TAGAGCAAATGGCTCCAG -3’

Ftoex1R 5’-CATCCACTCAGCCATCC-3’

Ftoex2F 5’-AAGCGTCTACCTTCAAGTTC-3’

Ftoex2R 5’-CCTAAGTGCAATTTGTACCC-3’

Ftoex3aF 5’-TGAAAGAAAACAGACTATTGG-3’

Ftoex3aR 5’-TTCTCTCTGACAGCCAGTTC-3’

Ftoex3bF 5’-GTACACAGAGGCTGAGATCG-3’

Ftoex3bR 5’-ACGAGTGTGCTTACTCATCC-3’

Ftoex4F 5’-TCCATATTTTATTGAGATACCAG-3’

Ftoex4R 5’-AATCACTACAAATTCAAAGTATCC-3’

Ftoex5F 5’-CTGTTCACCTTTTGTTCCAG-3’

Ftoex5R 5’-CAATAAACTGTCCACCCAAC-3’

Ftoex6F 5’-ATGTACAGCTGGAAGAGTGC-3’

Ftoex6R 5’-TCCCCTCTGACTAGGATCTC-3’

Ftoex7F 5’-GGAAGCCATTGTCCTTG-3’

Ftoex7R 5’-TTGGACGACGTATACATTTG-3’

Ftoex8F 5’-TGTCCTTATTTGCTTATGGG-3’

Ftoex8R 5’-CAACCTTAGTTCTCTGAGCG-3’

Ftoex9aF 5’-TACTCCTGTGCGCTAGTCAG-3’

Ftoex9aR 5’AAAGAGGAGAGCAGGTGAG-3’

Ftoex9bF 5’-ttcaccttccaggctactc-3’

Ftoex9bR 5’-ACCCAGGTCCTATAACCACT-3’

**Mutant Construct Production**

Primers used to generate mutant constructs (bold codons indicate mutation sites):

hfto2F: 5’-CATCATCATCATCACAAACGTACCCCGACCGCG-3’

hfto2R: 5’-CGCGGTCGGGGTACGTTTGTGATGATGATGATG-3’

ChftoF: 5’-GCGTTGCAGGATGTAATCGTATGATGATGATGATGGC-3’

ChftoR: 5’-GCCATCATCATCATCATACGATTACATCCTGCAACGC-3’

CmftoF: 5’-GCCATCATCATCATCATCACgattatatcttagaacGC-3’

CmftoR: 5’-GCGTTCTAAGATATAATCGTGATGATGATGATGATGGC-3’

1-D329mftoF 5’-caggcaccttggat**tag**atcttagaacgctg-3’

1-D329mftoR 5’-gcgttctaagat**cta**atccaaggtgcctgttg-3’

1-k387mftoF 5’-ggcagtcgatacaaa**tag**tgcaccgattggtgg-3’

1-k387mftoR 5’-ccaatcggtgca**cta**tttgtatcgactgccttg-3’

1-e408mftoF 5’-ggaagaagatggag**tga**gtgacaaatgcggtgc3’

1-e408mftoR 5’-ccgcatttgtcac**tca**ctccatcttcttccac-3’

I367FmftoF: 5’-caaggagaggaa**ttc**cataatgaggtggag-3’

I367FmftoR: 5’-ccacctcattatg**gaa**ttcctctccttgtttc-3’

I367AmftoF: 5’-CAAGGAGAGAGGAA**GCT**CATAATGAGGTGGAG-3’

I367AmftoR: 5’-CCACCTCATTATG**AGC**TTCCTCTCCTTGTTTC-3’

**Protein Extraction and Immunoblotting**

Mouse brain and liver tissue samples were homogenized using ceramic beads and a Precellys automated homogeniser (Stretton Scientific Ltd, Derbyshire) and lysed in CelLytic MT Cell Lysis Reagent (Sigma) with 1x complete protease inhibitor cocktail (Sigma). Protein concentrations were determined using the Bradford protein assay measured at 595 nm (Sigma). Protein samples were heated for 10 minutes at 70°C in sample buffer (final concentration of 30 mM Tris-HCl [pH 6.8], 5% glycerol, 0.005% bromophenol blue, 2.5% β-mercaptoethanol, 100mM Dithiotreitol (DTT).

Protein lysates (~40μg) were separated using a denaturing SDS-PAGE 7-15% Tris-HCl BioRad acrylamide gel (BioRad) alongside a “SeeBlue” (Invitrogen) protein ladder. The gel was then blotted using a Transblot SD system (BioRad) onto polyvinylidene difluoride (PVDF) membrane (Hybond-P Amersham). Membranes were blocked overnight at 4^o^C with 5% skimmed milk powder, 0.1% Triton X-100 in TBS. Membranes were probed with primary (1:500; rabbit anti-FTO, Eurogentec, Custom Antibody against recombinant mFTO protein) and secondary goat-anti rabbit (1:1000; BioRad) for 1 hour at room temperature. Loading control β-actin (1:1000; Santa Cruz). Enhanced Chemiluminescence Plus (ECL plus; Amersham) was used according to manufacturer’s instructions to allow visualization of protein on ECL film, developed using the Compact X4 (Xograph).

**Cell culture and confocal microscopy**

COS-7 cells (passage number 11-12) were grown in Dulbecco's Modified Eagle Medium (D-MEM, 4500 mg/L D-Glucose; Gibco BRL, Invitrogen) supplemented with 10% (v/v) foetal calf serum, 100 U/ml penicillin and 100 μg/ml streptomycin in a humidified 5% CO_2_ atmosphere. 2x10^5^ cells were plated onto glass cover slips one day before transient transfection. Two days after transfection, live cells were fixed for 15 minutes with 3.7% formaldehyde and permeabilized for five minutes in ice-cold acetone before mounting on glass slides using Vectashield hardset mounting medium with 4',6-diamidino-2-phenylindole (DAPI, VECTOR Laboratories).

PC-12 cells (passage number 5-7) were cultured in collagen coated dishes at 37 °C, 5% CO_2_ in RPMI 1640 supplemented with 10%, foetal calf serum and antibiotics. 24 hours before transfection cells were plated on fluorodishes (World Precision Instruments, USA) at 90-95% confluency. The PC-12 cells were fixed in ice-cold methanol. They were then permeabilized with 0.1% (w/v) Triton-X in PBS. A polyclonal rabbit anti-FTO antibody was diluted 1 in 100 in PBST and incubated for one hour. After washing a CY5 (ZyMax Grade) goat anti-rabbit secondary antibody diluted 1 in 100 was incubated for one hour. Cells were washed and slides mounted with Vectashield containing DAPI (Vector Laboratories, USA) according to the manufacturer’s instructions.

A COS-7 cell line was transfected with YFP-Fto, YFP-Fto^I367F^ or YFP constructs, using the TransIT-LT1 transfection reagent (Mirus, Cambridge BioScience). The PC-12 cell line was transfected with YFP-Fto, YFP-Fto^I367F^ or YFP constructs, using the Lipofectamine 2000 transfection reagent (Invitrogen). COS-7 cells expressing YFP-Fto or YFP were visualized by confocal imaging using an LSM 510 META confocal microscope and a Plan-Apochromat 63x/1.4 oil objective (Carl Zeiss). YFP was excited using the 514 nm line of the Argon laser and emitted light was collected between 522 and 565 nm in the Meta channel of the confocal system. Cy5 was excited at 633 nm with light from a HeliumNeon laser, and emission was detected in the Meta channel at wavelengths >640 nm. DAPI was excited in the two-photon mode with the 740 nm line of a Chameleon laser and emitted light detected between 437 and 480 nm in the Meta channel.

**Microarray Analysis**

**RNA extraction and microarray sample preparation -** Total ribonucleic acid (RNA) from free fed animals was prepared from gastrocnemius skeletal muscle, liver, and abdominal white adipose tissue (WAT) of wild-type (n = 4) and I367F (Heterozygous n = 3, homozygous n = 4 for microarray. Interscapular brown adipose tissue (BAT) was prepared for qRT-PCR. RNA was purified using an RNeasy Mini Kit plus for liver, RNeasy fibrous Mini Kit RNeasy for skeletal muscle or Lipid Tissue Mini Kit for white and brown adipose tissue (Qiagen, USA) following the manufacturer’s protocol. Hypothalamus tissue was prepared from whole brain by dissection, RNA was prepared using an RNeasy Plus Mini Kit (Qiagen, USA) for qRT-PCR. RNA concentration and integrity were assessed using a NanoDrop spectrophotometer and an Agilent 2100 BioAnalyser respectively.

Samples were prepared for Affymetrix analysis from tissues derived from wild-type, heterozygous and homozygous *FTO^I367F^* mice. The starting RNA was 300ng for liver and skeletal muscle and 600ng for abdominal white adipose tissue. The latter was prepared as 2 x 300ng starting RNA amounts and pooled at the cRNA stage. The Affymetrix GeneChip whole transcript (WT) sense target labelling kit (P/N901178) was used according to protocol Rev.1 of the manual. 3 μg of fragmented ssDNA was hybridised to Mouse Gene 1.0 ST arrays. Samples were washed and stained on the GeneChip Fluidics Station 450 using the FS450_0007 fluidics protocol. Arrays were scanned on an Affymetrix GeneChip 3000 7G scanner.

**Data processing -** Microarray data were RMA normalised independently for each tissue using GeneSpring GX10.0 (Agilent). Differentially expressed genes were identified using an unpaired t-test with a p value cut off of ≤0.05 and a fold change difference between wild-type and mutant FTO of ≥1.5.

**Global pathway analysis -** GenMAPP 2.1 [1] and MAPPFinder 2.1 were employed to assess significantly regulated gene ontology (GO) terms in the data sets described above. At least 3 genes and ≥20% genes in a GO term needed to be changing with a permuted P value of ≤0.05 to be included. The Ingenuity Pathways Analysis tool (Ingenuity Systems, www.ingenuity.com) was used to gain additional pathways information. Canonical pathways analysis identified the pathways from the Ingenuity Pathways Analysis library of canonical pathways that were most significant to the data sets of statistically changing genes derived from GeneSpring. Genes from the data set that met a ≥1.5 fold (WAT and skeletal muscle) or ≥1.3 fold (liver) cutoff and were associated with a canonical pathway were considered for the analysis.

**Clustering of pathways genes** – Immune and fatty acid metabolism genes from Ingenuity and or GenMAPP pathways were imported into GeneSpring GX10.0 and clustered using the hierarchical, Euclidean, centroid algorithm. Inflammation GO terms, showing significantly altered gene expression in 16 week white adipose tissue, were derived from GeneSpring.

**qRT-PCR -** The extracted RNA was stored at -80°C. cDNA was prepared using superscript III reverse transcriptase (Invitrogen) according to the manufacturer’s instructions. Separate quantitative PCR was performed using SYBR Green, or TaqMan Gene Expression Assay reagents and TaqMan FAM dye-labelled probes (Applied Biosystems Inc, USA) using an ABIPRISM 7700 Sequence detector (Perkin Elmer, Boston, MA). Taqman Assay ID for each Taqman probe are given in the Supplementary Table 4. For SYBR green detection, primers used were as described [2]. The expression of *Npy*, *Agrp*, and *Pomc* were assessed by SYBR green detection as described [3]. All data was normalized to expression of the endogenous house keeping gene glyceraldehyde 3-phosphate dehydrogenase (GAPDH) and analyzed by the Comparative ΔΔC_T_ method to determine the difference in sample groups relative to control animals.

**Intraperitoneal Glucose Tolerance Test.**

Each mouse was fasted overnight to establish a baseline glucose level "T0" (time zero). Mice were weighed, and a blood sample was collected from the tail vein after administration of local anesthesia (Emla cream) using Lithium-Heparin microvette tubes (Sarstedt). The mouse then received an intraperitoneal injection of 2 g glucose/kg body wt (20% glucose in 0.9% NaCl). Subsequent blood samples were taken (60 and 120 min or 10, 20 and 30min) after injection. Plasma was obtained from whole blood by centrifugation (3000 rpm x 10 min). Plasma glucose was measured using an Analox Glucose Analyser GM9.

**Liver Triglyceride Quantification Kit.**

Liver triglyceride concentrations were measured at 24 weeks using a colorimetric triglyceride quantification assay kit (Abcam Plc, Cambridge).

**Supplementary References**

1. Salomonis N, Hanspers K, Zambon AC, Vranizan K, Lawlor SC, et al. (2007) GenMAPP 2: new features and resources for pathway analysis. BMC Bioinformatics 8: 217.

2. Girard CA, Wunderlich FT, Shimomura K, Collins S, Kaizik S, et al. (2008) Expression of an activating mutation in the gene encoding the KATP channel subunit Kir6.2 in mouse pancreatic beta cells recapitulates neonatal diabetes. J Clin Invest. 119(1):80-90. doi: 10.1172/JCI35772.

3. Piper ML, Unger EK, Myers MG, Jr., Xu AW (2008) Specific physiological roles for signal transducer and activator of transcription 3 in leptin receptor-expressing neurons. Mol Endocrinol 22: 751-759.

4. Geoghegan KF, Dixon HB, Rosner PJ, Hoth LR, Lanzetti AJ, et al. (1999) Spontaneous alpha-N-6-phosphogluconoylation of a "His tag" in Escherichia coli: the cause of extra mass of 258 or 178 Da in fusion proteins. Anal Biochem 267: 169-184.
